# Analysis of Variance Components for Genetic Markers with Unphased Genotypes

**DOI:** 10.3389/fgene.2016.00123

**Published:** 2016-07-13

**Authors:** Tao Wang

**Affiliations:** Division of Biostatistics, Institute for Health and Society, Medical College of WisconsinMilwaukee, WI, USA

**Keywords:** Fisher's ANOVA model, analysis of variance, general linear model, general multi-allelic model, genetic variance components, orthogonal partition, allelic interactions, least square estimates

## Abstract

An ANOVA type general multi-allele (GMA) model was proposed in Wang (2014) on analysis of variance components for quantitative trait loci or genetic markers with phased or unphased genotypes. In this study, by applying the GMA model, we further examine estimation of the genetic variance components for genetic markers with unphased genotypes based on a random sample from a study population. In one locus and two loci cases, we first derive the least square estimates (LSE) of model parameters in fitting the GMA model. Then we construct estimators of the genetic variance components for one marker locus in a Hardy-Weinberg disequilibrium population and two marker loci in an equilibrium population. Meanwhile, we explore the difference between the classical general linear model (GLM) and GMA based approaches in association analysis of genetic markers with quantitative traits. We show that the GMA model can retain the same partition on the genetic variance components as the traditional Fisher's ANOVA model, while the GLM cannot. We clarify that the standard F-statistics based on the partial reductions in sums of squares from GLM for testing the fixed allelic effects could be inadequate for testing the existence of the variance component when allelic interactions are present. We point out that the GMA model can reduce the confounding between the allelic effects and allelic interactions at least for independent alleles. As a result, the GMA model could be more beneficial than GLM for detecting allelic interactions.

## 1. Introduction

Typically, there are two different ways in assessing the statistical association of a categorical variable with a continuous outcome. We can either make a direct comparison of the group means among groups defined by the categorical variable or assess the variation that the categorical variable may contribute to the total variance of the continuous outcome. The former approach is usually conducted via fitting a general linear model (GLM) with or without an adjustment for other covariates. The latter approach is referred as the analysis of variance (ANOVA), which examines a quantitative outcome variable by partitioning its total variance into variance components attributable to different sources of variation. The original ANOVA model was proposed initially by Fisher (1918) and formalized later in Fisher (1925). The traditional ANOVA approach on estimation of variance components was mainly based on Henderson's method I-III by equating the observed sums of squares to their expected values (Henderson, 1953). Currently, for predictors with observed group levels, their variance components can be estimated via ANOVA tables, which are mostly based on the sequential (Type I) sums of squares or partial reductions in (Type III) sums of squares via fitting GLM (Searle, 1971; Searle et al., 1992). For predictors with observed or unobserved group levels, their variance components could also be evaluated via maximum likelihood estimation (MLE) or restricted maximum likelihood (REML) (see Searle et al., 1992).

In genetic association studies, the standard GLM or ANOVA approaches may not be directly applicable to genetic markers with unphased genotypes. In humans, a marker genotype is a combination of one paternal and one maternal allele. But the phase information (i.e., the origin of parental alleles) is often missing from most of the current genotype typing technologies. Appropriate modification on the classical GLM or ANOVA methods is therefore needed in order to overcome this unknown phase problem. In Wang (2011), we introduced several coding schemes for unphased marker genotypes in constructing the dummy-variable based GLM. In Zeng et al. (2005) and Wang (2014), some revised Fisher's ANOVA models were also proposed for ANOVA analysis of quantitative trait loci or genetic markers with unphased genotypes, which were referred as the general biallelic (G2A) or general multi-allelic (GMA) models. The estimation of genetic variance components based on the G2A and GMA models were also briefly explored.

In this study, by applying the GMA model, we further examine estimation of genetic variance components for genetic markers with unphased genotypes from a random sample of individuals in a study population. First, for a single locus GMA model, we derive the least square estimates (LSE) of model parameters in fitting the GMA model based on the genotypic group means and allele frequency estimates. Than we construct estimators of the variance components for one marker in Hardy-Weinberg disequilibrium (HWD). Next, we consider a fully parameterized two-locus GMA model. We derive the LSE of model parameters in fitting the GMA model and develop estimators of the variance components for two genetic markers in an equilibrium population. In both one locus and two loci cases, we also explore the difference between the GLM and GMA in association analysis of genetic markers. We show that the GMA models can provide the same partition on the genetic variance components as the original Fisher's ANOVA models but the GLM cannot. We clarify that the F-statistics based on the partial reductions in sums of squares from the standard ANOVA table for association testing of the fixed allelic effects in a GLM could be inadequate for testing the existence of variance components when higher order fixed allelic interactions are present. In addition, we point out that the GMA model can reduce the confounding between allelic effects and allelic interactions at least when the inheritance of alleles are independent. As the result, the GMA model could be more beneficial than GLM for detecting allelic interactions. Finally, a simulation example is presented to show the difference between GLM and GMA models on partitioning variance components. The performance in model selection between using GLM and GMA models is also examined.

## 2. Models and results

Consider a random sample of size *N* from a study population. Let *y*_*i*_, *i* = 1, … , *N*, be their observed phenotypic values for a quantitative trait *Y*, and *g*_*i*_, *i* = 1, … , *N*, be their observed unphased genotypes at certain genetic marker loci. The quantitative trait *Y* is assumed to be affected by both genetic and environmental effects. Let *G* denote the true (unobservable) genotypic value, which could be affected by many genetic factors. If we ignore the genetic by environmental interactions, the relationship between the quantitative trait and marker genotypes can be modeled via a GLM

(1) $$y_{i}=\beta z_{i}+\text{E}(G|g_{i})+\epsilon_{i},\;i=1,\ldots ,N,$$

where *z*_*i*_ is a vector of the adjusted environmental covariates with fixed effects β, E(*G*|*g*_*i*_) represents the expected genotypic value of the *i*-th individual given his/her observed marker genotypes *g*_*i*_, and ϵ_*i*_ is a model residual error contributed by other environmental and genetic factors that cannot be captured by the covariates *z*_*i*_ and marker genotypes *g*_*i*_. We also assume that ϵ_*i*_, *i* = 1, … , *N*, are independent and identically distributed (i.i.d) with E(ϵ_*i*_) = 0 and V(ϵ_*i*_) = *V*_ϵ_. Besides, {ϵ_*i*_, *i* = 1, … , *N*} are independent of {*g*_*i*_, *i* = 1, … , *N*}. Then, the total phenotypic variance *V*_*Y*_ = *V*(E(*G*|*g*)) + E(*V*(*G*|*g*)) = *V*(E(*G*|*g*)) + *V*_ϵ_. In the rest of the paper, we focus on comparing the GLM and GMA modeling on the expected genotypic values E(*G*|*g*) and assessing the genetic variance components contributed by the allelic effects and allelic interactions at the marker loci to the expected genotypic variance *V*(E(*G*|*g*)).

### 2.1. One-locus models

Consider a single marker locus with multiple alleles *A*_1_, …, *A*_*m*_ (*m* ≥ 2). Let *p*_*j*_, *j* = 1, … , *m*, be the allele frequencies ($\overset{m}{\underset{j=1}{\sum }}p_{j}=1$), and *p*_*jk*_ = *P*(*A*_*j*_*A*_*k*_), *j, k* = 1, … , *m* (*j* ≤ *k*), be the genotype frequencies ($\underset{j\leq k}{\sum }p_{jk}=1$), in a study population. For a random sample of size *N* from the study population, suppose that there are *n*_*jk*_ individuals carrying genotypes *A*_*j*_*A*_*k*_ for *j* ≤ *k*, *j, k* = 1, … , *m* ($N=\overset{m}{\underset{j=1}{\sum }}\overset{m}{\underset{k=j}{\sum }}n_{jk}$). For notation simplicity, we also let *p*_*kj*_ = *p*_*jk*_ and *n*_*kj*_ = *n*_*jk*_ for *k* > *j*, *j, k* = 1, … , *m*. Then, $p_{j}=p_{jj}+\underset{k\neq j}{\sum }p_{jk}/2$, for *j* = 1, … , *m*. Let $n_{j\cdot }=2n_{jj}+\underset{k\neq j}{\sum }n_{jk}$, which is the total number of alleles *A*_*j*_ carried by the sampled individuals (*j* = 1, … , *m*). Assume that the observed marker genotypes *g*_*i*_, *i* = 1, … , *N*, are i.i.d. and follow a multinomial distribution. Then the MLE of the genotype frequencies are given by ${\hat{p}}_{jk}=n_{jk}/N$, and the MLE of the allele frequencies are given by ${\hat{p}}_{j}=n_{j\cdot }/\left(2N\right)={\hat{p}}_{jj}+\underset{k\neq j}{\sum }{\hat{p}}_{jk}/2$, for *j* = 1, … , *m*. Also, let *y*_*jki*_,*i* = 1, … , *n*_*jk*_ be the observed phenotypic values of the individuals carrying genotypes *A*_*j*_*A*_*k*_. We define the observed genotypic group means ${ȳ}_{jk\cdot }={ȳ}_{kj\cdot }=\overset{n_{jk}}{\underset{i=1}{\sum }}y_{jki}/n_{jk}$ for *j, k* = 1, … , *m*, the allele averaged means ${ȳ}_{j\cdot \cdot }={ȳ}_{\cdot j\cdot }=\left(2n_{jj}{ȳ}_{jj\cdot }+\underset{k\neq j}{\sum }n_{jk}{ȳ}_{jk\cdot }\right)/n_{j\cdot }$ for *j* = 1, … , *m*, and the grand mean ${ȳ}_{\cdot \cdot \cdot }=\overset{m}{\underset{j=1}{\sum }}\overset{m}{\underset{k=j}{\sum }}n_{jk}{ȳ}_{jk\cdot }/N$. In addition, we define the allele weighted means ${ȳ}_{j\cdot }^{*}={ȳ}_{\cdot j}^{*}=\overset{m}{\underset{k=1}{\sum }}{\hat{p}}_{k}{ȳ}_{jk\cdot }$ for *j* = 1, … , *m*, and the weighted overall mean ${ȳ}_{\cdot \cdot }^{*}=\overset{m}{\underset{j=1}{\sum }}\overset{m}{\underset{k=1}{\sum }}{\hat{p}}_{j}{\hat{p}}_{k}{ȳ}_{jk\cdot }=\overset{m}{\underset{j=1}{\sum }}{\hat{p}}_{j}^{2}{ȳ}_{jj\cdot }+\overset{m-1}{\underset{j=1}{\sum }}\overset{m}{\underset{k=j+1}{\sum }}2{\hat{p}}_{j}{\hat{p}}_{k}{ȳ}_{jk\cdot }$. Note that in a classical two-way factorial ANOVA model, each individual can receive one and only one level assignment from each of the two factors. At a marker locus, however, an individuals can carry a homozygous genotype “*A*_*j*_*A*_*j*_” (*j* = 1, … , *m*) with two *A*_*j*_ alleles indistinguishable. In general, the allele weighted means ${ȳ}_{j\cdot }^{*}$ may not be the same as the allele averaged means ȳ_*j*··_, and the weighted overall mean ${ȳ}_{\cdot \cdot }^{*}$ may also differ from the grand mean ȳ_···_.

To assess the allelic effects on the expected genotypic values (or phenotypic values), let us first take a brief review of Fisher's one-locus ANOVA model (see Kempthorne, 1957; Weir and Cockerham, 1977). Since the marker genotypes are unphased, we usually assume that the paternal and maternal gametes share the same set of alleles, have the same allele frequencies, and contribute the same allelic effects at the marker locus. The fact that each allele contributes the same genetic effect regardless of its parental origins implies that the expected genotypic values *G*_*jk*_ = E(*G*|*g* = *A*_*j*_*A*_*k*_) should satisfy the symmetric property: *G*_*jk*_ = *G*_*kj*_, for *j* ≠ *k*. So there are totally *m*(*m* + 1)/2 possible distinctive expected genotypic values *G*_*jk*_, *j, k* = 1, …, *m*. By treating the paternal and maternal gametes as two independent risk factors, the traditional Fisher's one-locus ANOVA model for the expected genotypic values *G*_*jk*_ can be written as

(2) $$G_{jk}=\mu +\alpha_{j}+\alpha_{k}+\delta_{jk},\;j,k=1,\ldots ,m,$$

where α_*j*_ is referred as the *average* additive effect of the paternal or maternal allele *A*_*j*_ (*j* = 1, …, *m*), and δ_*jk*_ the *average* allelic interaction between two parental alleles *A*_*j*_ and *A*_*k*_ (*j, k* = 1, …, *m*). It is known that not all the parameters in the above model are estimable due to an over-parameterization of the model on the expected genotypic values. Typically, the following constraints can be added on the model parameters (see Kempthorne, 1957).

(3) $$\sum_{j=1}^{m}p_{j}\alpha_{j}=0,\;\sum_{j=1}^{m}p_{j}\delta_{jk}=0\text{ for }k=1,\ldots ,m.$$

From the symmetric property of *G*_*jk*_, we also assume that δ_*jk*_ = δ_*kj*_, for *j* ≠ *k*. With these constraints, if we further incorporate model (2) into GLM (1) and ignore the adjusted covariates, the LSE of parameters are given by $\hat{\mu }={ȳ}_{\cdot \cdot }^{*}$, ${\hat{\alpha }}_{j}={ȳ}_{j\cdot }^{*}-{ȳ}_{\cdot \cdot }^{*}$ for *j* = 1, … , *m*, and ${\hat{\delta }}_{jk}={ȳ}_{jk\cdot }-{ȳ}_{j\cdot }^{*}-{ȳ}_{k\cdot }^{*}+{ȳ}_{\cdot \cdot }^{*}$ for *j, k* = 1, … , *m*. It has been known that, under Hardy-Weinberg equilibrium (HWE), the above model (2) can also provide an orthogonal partition of the expected genotypic variance as *V*(E(*G*|*g*)) = *V*_*A*_ + *V*_*D*_, where $V_{A}=2\underset{j}{\sum }p_{j}\alpha_{j}^{2}$ and $V_{D}=\underset{j,k}{\sum }p_{j}p_{k}\delta_{jk}^{2}$ are the so-called additive and dominance variance components. Weir and Cockerham (1977) also explored model (2) on partitioning the expected genotypic variance in HWD. In practice, as pointed out in Wang (2014), the symmetric property of δ_*jk*_'s and the irregular constraints (3) could make it difficult to fit model (2) using standard statistical software especially when the adjusted covariates are involved. Besides, the random variables that are responsible for the additive and dominance variance components are not explicitly defined in model (2).

The expected genotypic values can also be modeled via a classical dummy-variable based GLM. As shown in Wang (2011), we can introduce the following indicator variables to describe the inheritance of the two parental alleles for each individual

$$\begin{array}{l} z_{1j}=\{\begin{array}{l} \begin{array}{l} 1,\text{ the inherited paternal allele is }A_{j}\\ 0,\text{ the inherited paternal allele is not }A_{j} \end{array} \end{array},\\ z_{2j}=\{\begin{array}{l} \begin{array}{l} 1,\text{ the inherited maternal allele is }A_{j}\\ 0,\text{ the inherited maternal allele is not }A_{j} \end{array} \end{array} \end{array}$$

for *j* = 1, …, *m*. Though *z*_1*j*_ and *z*_2*j*_ cannot be defined on unphased heterozygous genotypes, we can define the following genotype coding variables for unphased genotypes

$$w_{j}(g)=z_{1j}+z_{2j}=\{\begin{array}{l} 2,\text{ if }g=A_{j}A_{j}\\ 1,\text{ if }g=A_{j}A_{j}^{c}\\ 0,\text{ if }g=A_{j}^{c}A_{j}^{c} \end{array}$$

for *j* = 1, …, *m*, and

$$\begin{array}{l} v_{jj}(g)=z_{1j}z_{2j}=\{\begin{array}{l} 1,\text{ if }g=A_{j}A_{j}\\ 0,\text{ otherwise} \end{array},\\ v_{jk}(g)=z_{1j}z_{2k}+z_{1k}z_{2j}=\{\begin{array}{l} 1,\text{ if }g=A_{j}A_{k}\\ 0,\text{ otherwise} \end{array} \end{array}$$

for *j* ≠ *k*, *j, k* = 1, …, *m*. Here, $A_{j}^{c}$ denotes any other allele rather than *A*_*j*_. By choosing *A*_*m*_ as a reference allele, we can construct the following GLM

(4) $$\text{E}(G|g_{i})=\mu_{0}+\sum_{j=1}^{m-1}a_{j}w_{j}(g_{i})+\sum_{j=1}^{m-1}\sum_{k=j}^{m-1}d_{jk}v_{jk}(g_{i}),$$

for *i* = 1, …, *N*, where *a*_*j*_ is usually referred as the *fixed* additive allelic effect of the paternal or maternal allele *A*_*j*_, and *d*_*jk*_ the *fixed* allelic interaction between two parental alleles *A*_*j*_ and *A*_*k*_, with respect to the reference allele *A*_*m*_. In terms of the expected genotypic values, we can show that μ_0_ = *G*_*mm*_, *a*_*j*_ = *G*_*jm*_ − *G*_*mm*_ and *d*_*jk*_ = (*G*_*jk*_ − *G*_*km*_) − (*G*_*jm*_ − *G*_*mm*_), for *j* = 1, …, *m* − 1 and *k* = *j*, …, *m* − 1.

Model (4) provides a full re-parameterization of the *m*(*m* + 1)/2 expected genotypic values. Suppose that there are no empty genotype groups for the observed random sample; i.e., *n*_*jk*_ > 0 for any *j, k* = 1, … , *m*. When we incorporate model (4) into GLM (1) and ignore the adjusted covariates, the LSE of the expected genotypic values are given by: Ĝ_*jk*_ = ȳ_*jk*_, for *j, k* = 1, … , *m* − 1 and *j* < *k*. Therefore, the LSE of parameters in model (4) can be easily derived in terms of the observed genotypic group means as ${\hat{\mu }}_{0}={ȳ}_{mm\cdot }$ and â_*j*_ = ȳ_*jm*·_ − ȳ_*mm*·_ for *j* = 1, … , *m* − 1, and ${\hat{d}}_{jk}={ȳ}_{jk\cdot }-{ȳ}_{jm\cdot }-{ȳ}_{km\cdot }+{ȳ}_{mm\cdot }$ for *j, k* = 1, … , *m* − 1 and *j* ≤ *k*. Note that these LSE could be sensitive to phenotypic outliers especially for small genotypic groups. For example, a few individuals may have genotype “*A*_*j*_*A*_*m*_” (j ≠ *m*) for a rare allele “*A*_*j*_.” If the genotypic group mean ȳ_*jm*·_ is much larger than ȳ_*mm*·_, the LSE â_*j*_ will be large. By choosing a common allele “*A*_*m*_” as the reference, we could improve the accuracy of LSE. Through incorporating model (4) into a GLM, we could also perform hypothesis tests on its model parameters via contrasts. In analysis of genetic variance components, however, it has been known that *w*_*j*_'s are often correlated with *v*_*jk*_'s as random variables over the random individuals even when the inheritance of paternal and maternal alleles are independent (Wang and Zeng, 2009). As the result, model (4) leads to a different partition of the expected genotypic variance *V*(E(*G*|*g*)) from the one defined in Fisher's ANOVA model (2).

To further dissect the confounding between the additive and dominance effects in model (4), we can make mean corrections on the indicator variables {*z*_1*i*_} and {*z*_2*j*_}, and introduce the following mean-corrected index variables (see Wang, 2014)

$$\begin{array}{l} x_{1j}(g)=z_{1j}(g)-\text{E}[z_{1j}(g)]=\{\begin{array}{ll} 1-p_{j}, & \text{the paternal allele is }A_{j}\\ -p_{j}, & \text{the paternal allele is not }A_{j} \end{array}\\ x_{2j}(g)=z_{2j}(g)-\text{E}[z_{2j}(g)]=\{\begin{array}{ll} 1-p_{j}, & \text{the maternal allele is }A_{j}\\ -p_{j}, & \text{the maternal allele is not }A_{j} \end{array} \end{array}$$

Then, we define the following modified genotype coding variables

$$\begin{array}{l} w_{j}^{*}(g)=x_{1j}+x_{2j}=\{\begin{array}{cc} 2(1-p_{j}), & \text{if }g=A_{j}A_{j}\\ 1-2p_{j}, & \text{if }g=A_{j}A_{j}^{c}\\ -2p_{j}, & \text{if }g=A_{j}^{c}A_{j}^{c} \end{array},\\ v_{jj}^{*}(g)=x_{1j}x_{2j}=\{\begin{array}{cc} {\left(1-p_{j}\right)}^{2}, & \text{if }g=A_{j}A_{j}\\ -p_{j}(1-p_{j}), & \text{if }g=A_{j}A_{j}^{c}\\ p_{j}^{2}, & \text{if }g=A_{j}^{c}A_{j}^{c} \end{array} \end{array}$$

for *j* = 1, … , *m*, and

$$v_{jk}^{*}(g)=x_{1j}x_{2k}+x_{1k}x_{2j}=\{\begin{array}{l} (1-p_{j})(1-p_{k})+p_{j}p_{k},\text{ if }g=A_{j}A_{k}\\ -2p_{k}(1-p_{j}),\text{ if }g=A_{j}A_{j}\\ -2p_{j}(1-p_{k}),\text{ if }g=A_{k}A_{k}\\ -p_{k}(1-2p_{j}),\text{ if }g=A_{j}A_{jk}^{c}\\ -p_{j}(1-2p_{k}),\text{ if }g=A_{k}A_{jk}^{c}\\ 2p_{i}p_{j},\text{ if }g=A_{jk}^{c}A_{jk}^{c} \end{array}$$

for *j* ≠ *k*, *j, k* = 1, …, *m*. Here, $A_{jk}^{c}$ denotes an allele which is different from both *A*_*j*_ and *A*_*k*_. Note that the modified genotype coding variables $w_{j}^{*}\left(g\right)=w_{j}\left(g\right)-2p_{j}$, $v_{jj}^{*}\left(g\right)=v_{jj}\left(g\right)-p_{j}w_{j}\left(g\right)+p_{j}^{2}$, and $v_{jk}^{*}\left(g\right)=v_{jk}\left(g\right)-p_{j}w_{k}\left(g\right)-p_{k}w_{j}\left(g\right)+2p_{j}p_{k}$ are well defined on unphased genotypes, although the mean-corrected index variables *x*_1*j*_, *x*_2*k*_ cannot be defined on unphased heterozygous genotypes. By choosing *A*_*m*_ as a reference allele, we can construct the following GMA model (Wang, 2014)

(5) $$\text{E}(G|g_{i})=\mu^{*}+\sum_{j=1}^{m-1}\alpha_{j}^{*}w_{j}^{*}(g_{i})+\sum_{j=1}^{m-1}\sum_{k=j}^{m-1}\delta_{jk}^{*}v_{jk}^{*}(g_{i}),$$

for *i* = 1, …, *N*. Still, in model (5), we refer the parameters $\alpha_{j}^{*}$ as the *average* additive allelic effects and $\delta_{jk}^{*}$ (*j* ≤ *k*) as the *average* allelic interactions, with respect to the reference allele *A*_*m*_. Both the GMA model (5) and the GLM (4) can provide a full parameterization of the expected genotypic values *G*_*jk*_. Comparing to model (4), one major advantage of model (5) is that it can retain the same partition on the genetic variance components as the one from Fisher's ANOVA model (2). In fact, under the constraints (3) plus the symmetric property of δ_*jk*_, model (2) can be re-written as

(6) $$\text{E}(G|g_{i})=\mu +\sum_{j=1}^{m}\alpha_{j}w_{j}^{*}(g_{i})+\sum_{j=1}^{m}\sum_{k=j}^{m}\delta_{jk}v_{jk}^{*}(g_{i}),$$

for *i* = 1, …, *N*. Model (5) is a simplified version of model (6) by further replacing the redundant variables $w_{m}^{*}$, $v_{jm}^{*}$ and $v_{mm}^{*}$ by $w_{m}^{*}=-\overset{m-1}{\underset{j=1}{\sum }}w_{j}^{*}$, $v_{jm}^{*}=-v_{jj}^{*}-\overset{m-1}{\underset{k=1}{\sum }}v_{jk}^{*}$ for *j* = 1, … , *m* − 1, and $v_{mm}^{*}=\overset{m-1}{\underset{j=1}{\sum }}\overset{m-1}{\underset{k=j}{\sum }}v_{jk}^{*}$. We can see that both models (5) and (2) share exactly the same additive and dominance variance components. They become equivalent when we take μ^*^ = μ, $\alpha_{j}^{*}=\alpha_{j}-\alpha_{m}$, and $\delta_{jk}^{*}=\delta_{jk}-\delta_{jm}-\delta_{km}+\delta_{mm}$, for *j, k* = 1, … , *m* − 1 and *j* ≤ *k*. Note that model (5) does not contain redundant parameters. Therefore, it does not require constraints on its model parameters. Besides, the random variables that are responsible for the additive and dominance variance components are explicitly defined in model (5). In practice, similar to enforcing the constraints (3) on model (2), we can create the variables $w_{j}^{*}$ and $v_{jk}^{*}$ by replacing the allele frequencies *p*_*j*_'s by their MLE ${\hat{p}}_{j}$'s. Then, by incorporating model (5) into GLM (1), we can treat these modified genotype coding variables as regular fixed covariates and fit the model using the ordinary LS approach. The hypothesis of *H*_0_:*V*_*A*_ = 0 for existence of the additive variance component can also be tested via testing $H_{0}:\alpha_{j}^{*}=0,j=1,\ldots ,m-1$ for the average additive allelic effects.

The GLM (4) and GMA model (5) can be transformed easily from one to the other. From the relationship between their genotype coding variables, we can show that

$$\{\begin{array}{l} \mu^{*}=\gamma +2\sum_{j=1}^{m-1}p_{j}a_{j}+\sum_{j=1}^{m-1}\sum_{k=1}^{m-1}p_{j}p_{k}d_{jk}\\ \alpha_{j}^{*}=a_{j}+\sum_{k=1}^{m-1}p_{k}d_{jk},\;j=1,\ldots ,m-1 \end{array}$$

and $\delta_{jk}^{*}=d_{jk}$ for *j* ≤ *k*, *j, k* = 1, … , *m* − 1. Here, for notation simplicity, we define *d*_*kj*_ = *d*_*jk*_, for *j* < *k*. To fit model (5), instead of solving its normal equations directly, we can derive the LSE of its model parameters from the LSE of model (4) as the following.

$$\{\begin{array}{l} {\hat{\mu }}^{*}=\sum_{j=1}^{m}{\hat{p}}_{j}^{2}{\bar{y}}_{jj\cdot }+\sum_{j=1}^{m-1}\sum_{k=j+1}^{m}2{\hat{p}}_{j}{\hat{p}}_{k}{\bar{y}}_{jk\cdot }={\bar{y}}_{\cdot \cdot }^{*}\\ {\hat{\alpha }}_{j}^{*}={\bar{y}}_{j\cdot }^{*}-{\bar{y}}_{m\cdot }^{*}\;,\;j=1,\ldots ,m-1 \end{array}$$

and ${\hat{\delta }}_{jk}^{*}={\hat{d}}_{jk}={ȳ}_{jk\cdot }-{ȳ}_{jm\cdot }-{ȳ}_{km\cdot }+{ȳ}_{mm\cdot }$ for *j* ≤ *k*, *j, k* = 1, … , *m* − 1. Note that the LSE of the average additive allelic effects are calculated from allele weighted means, which could be more robust to phenotypic outliers than the LSE of fixed additive allelic effects. It is also interesting to see that both the fixed additive allelic effects *a*_*j*_'s and the fixed allelic interactions *d*_*jk*_'s may affect the average additive allelic effects $\alpha_{j}^{*}$'s, though the fixed allelic interactions keep the same as the average allelic interactions. In general, the hypothesis test of *H*_0_ : *a*_*j*_ = 0, *j* = 1, … , *m* − 1, for the fixed additive effects in a GLM (4) is not equivalent to the hypothesis test of $H_{0}:\alpha_{j}^{*}=0,j=1,\ldots \;,m-1$, for the average additive effects in an equivalent GMA model (5). Therefore, the standard F-statistics for testing the fixed additive effects in model (4) could be inadequate for testing the existence of the additive variance component *V*_*A*_ when significant fixed (or average) allelic interactions are present.

In ANOVA, we treat {*x*_1*j*_, *j* = 1, … , *m* − 1} and {*x*_2*k*_, *k* = 1, … , *m* − 1} as random variables over the sampled individuals. Model (5) provides an approximation to the expected genotypic values E(*G*|*g*) by a linear combination of the random variables *x*_1*j*_, *j* = 1, … , *m* − 1 and *x*_2*k*_, *k* = 1, … , *m* − 1, plus their cross-product terms for allelic interactions. To model the genotypic distributions, let $g_{i}^{*}=\left(z_{1j}\left(g_{i}\right)z_{2k}\left(g_{i}\right),j,k=1,\ldots \;,m,j\leq k\right)$ be a vector that describes the unphased genotypic categories for *i* = 1, … , *N*. As usual, we assume that the marker genotypes $g_{1}^{*},\ldots \;,g_{N}^{*}$ are i.i.d. from a multinomial distribution of *Mult*(1, {*p*_*jk*_, *j, k* = 1, … , *m, j* ≤ *k*}). Under this assumption, we can show that the vectors *g*_1*i*_ = (*z*_11_(*g*_*i*_), … , *z*_1*m*_(*g*_*i*_)), *i* = 1, … , *N*, of paternal allele indicator variables are i.i.d. from *Mult*(1, {*p*_1_, … , *p*_*m*_}); and the vectors *g*_2*i*_ = (*z*_21_(*g*_*i*_), … , *z*_2*m*_(*g*_*i*_)), *i* = 1, … , *N*, of maternal allele indicator variables are also i.i.d. from *Mult*(1, {*p*_1_, … , *p*_*m*_}). When the inheritance of paternal and maternal alleles are independent, the study population is under HWE and the mean corrected index variables {*x*_1*j*_(*g*_*i*_), *j* = 1, … , *m*} are independent of {*x*_2*j*_(*g*_*i*_), *j* = 1, … , *m*} for any randomly sampled individual *i*. In HWD, however, the mean corrected index variables {*x*_1*j*_, *j* = 1, … , *m*} could be correlated with {*x*_2*j*_, *j* = 1, … , *m*}. Let us define

$$\{\begin{array}{l} D_{jj}=\text{Cov}(z_{1j},z_{2j})=\text{E}(x_{1j}x_{2j})=\text{E}(v_{jj}^{*})=p_{jj}-p_{j}^{2}\\ D_{jk}=\text{E}(x_{1j}x_{2k}+x_{1k}x_{2j})/2=\text{E}(v_{jk}^{*})/2=p_{jk}/2-p_{j}p_{k}. \end{array}$$

for *j* ≠ *k*, *j, k* = 1, … , *m*. Then, {*D*_*jk*_, *j, k* = 1, … , *m*} measure the departures from HWE, which satisfy $\overset{m}{\underset{j=1}{\sum }}D_{jk}=\overset{m}{\underset{k=1}{\sum }}D_{jk}=0$. From the observed genotypes, we have MLE ${\hat{D}}_{jj}=n_{jj}/N-{\hat{p}}_{j}^{2}$ for *j* = 1, … , *m*, and ${\hat{D}}_{jk}=n_{jk}/\left(2N\right)-{\hat{p}}_{j}{\hat{p}}_{k}$ for *j, k* = 1, … , *m* and *j* ≠ *k*.

It is more convenient to use GMA model (5) rather than GLM (4) for estimation of genetic variance components. In model (5), let $A=\overset{m-1}{\underset{j=1}{\sum }}\alpha_{j}^{*}w_{j}^{*}\left(g\right)$ and $D=\overset{m-1}{\underset{j=1}{\sum }}\overset{m-1}{\underset{k=j}{\sum }}\delta_{jk}^{*}v_{jk}^{*}\left(g\right)$ represent the additive and dominance components, respectively. In general, we can partition the expected genotypic variance as *V*(E(*G*|*g*)) = *V*_*A*_ + *V*_*D*_ + 2Cov(*A, D*). In Wang (2014), we have derived formulas for estimating the variance components *V*_*A*_, *V*_*D*_ and covariance component Cov(*A, D*) based on the parameters in model (5). By plugging in LSE of the parameters, we obtain estimators of the variance components *V*_*A*_, *V*_*D*_ and the covariance component Cov(*A, D*) as shown in Appendix A in Supplementary Material. It should be pointed out that these estimators of the variance and covariance components are different from the traditional ANOVA estimators when the marker genotypes are in HWD. Unlike the ANOVA estimators of variance components which could be negative when data are unbalanced, our estimators of the variance components are guaranteed to be non-negative. Similar to the ANOVA estimators, when the model residuals are normally distributed, these estimators become MLE of the variance and covariance components and likely possess the asymptotic normality property (see Searle, 1995). Meanwhile, with variations from both the genotypic group means and the MLE estimates of allele frequencies, these estimators are only asymptotically unbiased, while the original ANOVA estimators are unbiased.

Under HWE, we would expect an orthogonal partition of the expected genotypic variance as *V*(E(*G*|*g*)) = *V*_*A*_ + *V*_*D*_. By taking ${\hat{D}}_{jk}=0$ for *j, k* = 1, … , *m* in Appendix A in Supplementary Material, we obtain the following estimators

$$\begin{array}{l} {\hat{V}}_{A}=2\sum_{j=1}^{m}{\hat{p}}_{j}{\left({\bar{y}}_{j\cdot }^{*}-{\bar{y}}_{\cdot \cdot }^{*}\right)}^{2},\\ {\hat{V}}_{D}=\sum_{j=1}^{m}\sum_{k=1}^{m}{\hat{p}}_{j}{\hat{p}}_{k}{\left({\bar{y}}_{jk\cdot }-{\bar{y}}_{\cdot \cdot }^{*}\right)}^{2}-2\sum_{j=1}^{m}{\hat{p}}_{j}{\left({\bar{y}}_{j\cdot }^{*}-{\bar{y}}_{\cdot \cdot }^{*}\right)}^{2} \end{array}$$

If we further incorporate model (5) into GLM (1) and ignore the adjusted covariates, the estimator of residual variance is given by ${\hat{V}}_{\epsilon }=\overset{m}{\underset{j=1}{\sum }}\overset{m}{\underset{k=j}{\sum }}\overset{n_{jk}}{\underset{i=1}{\sum }}{\left(y_{jki}-{ȳ}_{jk\cdot }\right)}^{2}/N$. The estimator of total phenotypic variance is given by ${\hat{V}}_{Y}=\overset{m}{\underset{j=1}{\sum }}\overset{m}{\underset{k=j}{\sum }}\overset{n_{jk}}{\underset{i=1}{\sum }}{\left(y_{jki}-{ȳ}_{\cdot \cdot \cdot }\right)}^{2}/N$. We can show that ${\hat{V}}_{Y}={\hat{V}}_{A}+{\hat{V}}_{D}+{\hat{V}}_{\epsilon }$. Note that when ${\hat{D}}_{jk}=0$ for *j, k* = 1, … , *m*, the allele weighted means and the allele averaged means become the same; i.e., ${ȳ}_{j\cdot }^{*}={ȳ}_{j\cdot \cdot }$ for *j* = 1, … , *m*. Besides, the weighted overall mean ${ȳ}_{\cdot \cdot }^{*}$ is the same as the grand mean ŷ_···_. However, due to possible sampling variations from the sampled individuals, under HWE we could still have some ${\hat{D}}_{jk}\neq 0$, *j, k* = 1, … , *m*, which may lead to a slight deviation from the orthogonal partition of the expected genotypic variance.

When ${\hat{D}}_{jk}=0$ for *j, k* = 1, … , *m*, another nice feature of the GMA model (5) is that the LSE $\left\{{\hat{\alpha }}_{j}^{*},j=1,\ldots \;,m-1\right\}$ of its main effects will keep the same in a reduced GMA model when we ignore the allelic interactions. Let us represent the full GMA model (5) in a matrix form with the design matrix $X=\left(1_{N},X_{\alpha^{*}},X_{\delta^{*}}\right)$, where 1_*N*_ is a *N* by 1 vector with all its elements being 1, $X_{\alpha^{*}}={\left(w_{1}^{*}\left(g_{i}\right),\ldots \;,w_{m-1}^{*}\left(g_{i}\right);i=1,\ldots \;,N\right)}_{N\times \left(m-1\right)}$ and $X_{\delta^{*}}={\left(v_{11}^{*}\left(g_{i}\right),\ldots \;,v_{1,m-1}^{*}\left(g_{i}\right),\ldots \;,v_{m-1,m-1}^{*}\left(g_{i}\right);i=1,\ldots \;,N\right)}_{N\times m\left(m-1\right)/2}$, which correspond to the main effects $\alpha^{*}=\left(\alpha_{1}^{*},\ldots \;,\alpha_{m-1}^{*}\right)$ and allelic interactions $\delta^{*}=\left(\delta_{11}^{*},\ldots \;,\delta_{1,m-1}^{*},\ldots \;,\delta_{m-1,m-1}^{*}\right)$, respectively. We can show that $X^{\prime }X=diag\left(N,X_{\alpha^{*}}^{\prime }X_{\alpha^{*}},X_{\delta^{*}}^{\prime }X_{\delta^{*}}\right)$, which is a block diagonal matrix when ${\hat{D}}_{jk}=0$, for *j, k* = 1, … , *m* (see proof in Appendix B in Supplementary Material). Therefore, the LSE ${\hat{\mu }}^{*}={ȳ}_{\cdot \cdot \cdot }$ and ${\hat{\alpha }}^{*}={\left(X_{\alpha^{*}}^{\prime }X_{\alpha^{*}}\right)}^{-1}X_{\alpha^{*}}^{\prime }Y$, which do not depend on $X_{\delta^{*}}$. In other words, ignoring the average allelic interactions in model (5) does not affect the LSE of the intercept and the average additive effects in this case. The GLM (4) does not have such a property. In a reduced GLM (4) without the fixed allelic interactions, the LSE of its additive effects are ${â}_{j}={\hat{\alpha }}_{j}^{*}={ȳ}_{j\cdot }^{*}-{ȳ}_{m\cdot }^{*}$ for *j* = 1, … , *m* − 1, while in a full GLM (4) the LSE become â_*j*_ = ȳ_*jm*·_ − ȳ_*mm*·_, for *j* = 1, … , *m* − 1.

We can also estimate the genetic variance components with an adjustment for other covariates. First, by incorporating the GMA model (5) into GLM (1), we can fit GLM (1) using the ordinary LS approach. Next, based on the fitted model, we can calculate the fitted additive and dominance components ${Â}_{i}=\overset{m-1}{\underset{j=1}{\sum }}{\hat{\alpha }}_{j}^{*}w_{i}\left(g_{i}\right)$ and ${\hat{D}}_{i}=\overset{m-1}{\underset{j=1}{\sum }}\overset{m-1}{\underset{k=j}{\sum }}{\hat{\delta }}_{jk}^{*}v_{jk}\left(g_{i}\right)$, for each individual *i* = 1, … , *N*. Then, we can estimate *V*_*A*_ and *V*_*D*_ as the sample variances of {Â_*i*_, *i* = 1, … , *N*} and $\left\{{\hat{D}}_{i},i=1,\ldots \;,N\right\}$, respectively. Meanwhile, the covariance component Cov(*A, D*) can be estimated as the sample covariance between {Â_*i*_, *i* = 1, … , *N*} and $\left\{{\hat{D}}_{i},i=1,\ldots \;,N\right\}$.

### 2.2. Two-locus models

We consider an extension of the previous one-locus models to two loci. Assume that marker 1 has alleles *A*_11_, …, *A*_1_*m*__1__ (*m*_1_ ≥ 2) with *p*_11_, … , *p*_1_*m*__1__ being the allele frequencies, and marker 2 has alleles *A*_21_, …, *A*_2_*m*__2__ (*m*_2_ ≥ 2) with *p*_21_, … , *p*_2_*m*__2__ being the allele frequencies. Let *p*_*jkrs*_ = *P*(*A*_1*j*_*A*_1*k*_, *A*_2*r*_*A*_2*s*_) denote the joint genotype frequencies at the two marker loci in a study population. For a random sample of size *N* from the study population, let *n*_*jkrs*_ be the number of individuals who carry unphased genotypes *A*_1*j*_*A*_1*k*_ (*j* ≤ *k*) at locus 1, and *A*_2*r*_*A*_2*s*_ (*r* ≤ *s*) at locus 2, for *j, k* = 1, … , *m*_1_ and *r, s* = 1, … , *m*_2_ ($N=\overset{m_{1}}{\underset{j=1}{\sum }}\overset{m_{1}}{\underset{k=j}{\sum }}\overset{m_{2}}{\underset{r=1}{\sum }}\overset{m_{2}}{\underset{s=r}{\sum }}n_{jkrs}$). Then, the MLE of genotype frequencies ${\hat{p}}_{jkrs}=n_{jkrs}/N$. Let *y*_*jkrs, i*_,*i* = 1, … , *n*_*jkrs*_, be the observed phenotypic values of individuals carrying the joint genotypes (*A*_1*j*_*A*_1*k*_, *A*_2*r*_*A*_2*s*_). We define the observed genotypic group means ${ȳ}_{jkrs\cdot }=\overset{n_{jkrs}}{\underset{i=1}{\sum }}y_{jkrs,i}/n_{jkrs}$, for *j, k* = 1, … , *m*_1_ and *r, s* = 1, …, *m*_2_. Without distinguishing the origin of parental alleles, we assume that *n*_*kjrs*_ = *n*_*jkrs*_ for *k* > *j*, *j, k* = 1, … , *m*_1_, and *n*_*jksr*_ = *n*_*jkrs*_ for *r* > *s*, *r, s* = 1, … , *m*_2_. Let $n_{jk\cdot \cdot }=\overset{m_{2}}{\underset{r=1}{\sum }}\overset{m_{2}}{\underset{s=r}{\sum }}n_{jkrs}$ for *j, k* = 1, … , *m*_1_, and $n_{\cdot \cdot rs}=\overset{m_{1}}{\underset{j=1}{\sum }}\overset{m_{1}}{\underset{k=j}{\sum }}n_{jkrs}$ for *r, s* = 1, … , *m*_2_. We have the MLE of allele frequencies ${\hat{p}}_{1j}=\left(2n_{jj\cdot \cdot }+\underset{k\neq j}{\sum }n_{jk\cdot \cdot }\right)/\left(2N\right)$ for *j* = 1, … , *m*_1_ at locus 1, and ${\hat{p}}_{2r}=\left(2n_{\cdot \cdot rr}+\underset{s\neq r}{\sum }n_{\cdot \cdot rs}\right)/\left(2N\right)$ for *r* = 1, … , *m*_2_ at locus 2. For notation simplicity, we define ȳ_*kjrs*·_ = ȳ_*jkrs*·_ for *k* > *j* (*j, k* = 1, … , *m*_1_) and ȳ_*jksr*·_ = ȳ_*jkrs*·_ for *r* > *s* (*r, s* = 1, … , *m*_2_). In addition, we define the weighted genotypic group means ${ȳ}_{j\cdot rs}^{*}={ȳ}_{\cdot jrs}^{*}=\overset{m_{1}}{\underset{k=1}{\sum }}{\hat{p}}_{1k}{ȳ}_{jkrs}$, ${ȳ}_{jkr\cdot }^{*}={ȳ}_{jk\cdot r}^{*}=\overset{m_{2}}{\underset{s=1}{\sum }}{\hat{p}}_{2s}{ȳ}_{jkrs}$, ${ȳ}_{jk\cdot \cdot }^{*}={ȳ}_{kj\cdot \cdot }^{*}=\overset{m_{2}}{\underset{r,s=1}{\sum }}{\hat{p}}_{2r}{\hat{p}}_{2s}{ȳ}_{jkrs}$, ${ȳ}_{\cdot \cdot rs}^{*}={ȳ}_{\cdot \cdot sr}^{*}=\overset{m_{1}}{\underset{j,k=1}{\sum }}{\hat{p}}_{1j}{\hat{p}}_{1k}{ȳ}_{jkrs}$, ${ȳ}_{j\cdot \cdot \cdot }^{*}={ȳ}_{\cdot j\cdot \cdot }^{*}=\overset{m_{1}}{\underset{k=1}{\sum }}\overset{m_{2}}{\underset{r,s=1}{\sum }}{\hat{p}}_{1k}{\hat{p}}_{2r}{\hat{p}}_{2s}{ȳ}_{jkrs}$, and ${ȳ}_{\cdot \cdot r\cdot }^{*}={ȳ}_{\cdot \cdot \cdot r}^{*}=\overset{m_{1}}{\underset{j,k=1}{\sum }}\overset{m_{2}}{\underset{s=1}{\sum }}{\hat{p}}_{1j}{\hat{p}}_{1k}{\hat{p}}_{2s}{ȳ}_{jkrs}$, for *j, k* = 1, … , *m*_1_ and *r, s* = 1, … , *m*_2_. The weighted overall mean ${ȳ}_{\cdot \cdot \cdot \cdot }^{*}=\overset{m_{1}}{\underset{j,k=1}{\sum }}\overset{m_{2}}{\underset{r,s=1}{\sum }}{\hat{p}}_{1j}{\hat{p}}_{1k}{\hat{p}}_{2r}{\hat{p}}_{2s}{ȳ}_{jkrs}$.

Let *G*_*jkrs*_ = E(*G*(*g*)|*g* = *A*_1*j*_*A*_1*k*_, *A*_2*r*_*A*_2*s*_) be the expected genotypic value of individuals with the joint genotypes (*A*_1*j*_*A*_1*k*_, *A*_2*r*_*A*_2*s*_), for *j, k* = 1, …, *m*_1_ and *r, s* = 1, …, *m*_2_. Without distinguishing the origin of parental alleles, we assume that {*G*_*jkrs*_} satisfy the symmetric properties: *G*_*jkrs*_ = *G*_*kjrs*_ = *G*_*jksr*_ = *G*_*kjsr*_, for *j* < *k* and *r* < *s*. In general, there are totally *m*_1_*m*_2_(*m*_1_ + 1)(*m*_2_ + 1)/4 possible distinctive expected genotypic values. By treating the paternal and maternal gametes as two independent risk factors, the Fisher's two-locus ANOVA model for the expected genotypic values *G*_*jkrs*_ can be written as (see Kempthorne, 1957)

(7) $$\begin{array}{l} G_{jkrs}=\mu +\alpha_{1j}+\alpha_{1k}+\delta_{1jk}+\alpha_{2r}+\alpha_{2s}+\delta_{2rs}+{\left(\alpha \alpha \right)}_{jr}\\ \;+{\left(\alpha \alpha \right)}_{js}+{\left(\alpha \alpha \right)}_{kr}+{\left(\alpha \alpha \right)}_{ks}+{\left(\alpha \delta \right)}_{j,rs}+{\left(\alpha \delta \right)}_{k,rs}\\ \;+{\left(\delta \alpha \right)}_{jk,r}+{\left(\delta \alpha \right)}_{jk,s}+{\left(\delta \delta \right)}_{jk,rs} \end{array}$$

for *j, k* = 1, …, *m*_1_ and *r, s* = 1, …, *m*_2_. The parameters α_1*j*_ and δ_1*jk*_ are referred as the average additive and dominance effects at locus 1, α_2*r*_ and δ_2*rs*_ the average additive and dominance effects at locus 2, (αα)_*jr*_ the additive by additive interactions, (αδ)_*j, rs*_ the additive by dominance interactions, (δα)_*jk, r*_ the dominance by additive interactions, and (δδ)_*jk, rs*_ the dominance by dominance interactions. Still, due to an over-parameterization of the model for the expected genotypic values, not all the model parameters are estimable. From the symmetric property of the expected genotypic values, we usually assume that δ_1*jk*_ = δ_1*kj*_, δ_2*rs*_ = δ_2*sr*_, (αδ)_*j, rs*_ = (αδ)_*j, sr*_, (δα)_*jk, r*_ = (δα)_*kj, r*_, (δδ)_*jk, rs*_ = (δδ)_*kj, rs*_ = (δδ)_*jk, sr*_. In addition, the following constraints need to be added on the model parameters (Gallais, 1974; Weir and Cockerham, 1977).

(8) $$\begin{array}{l} \sum_{j=1}^{m_{1}}p_{1j}\alpha_{1j}=0,\;\sum_{r=1}^{m_{2}}p_{2r}\alpha_{2r}=0,\;\sum_{j=1}^{m_{1}}p_{1j}\delta_{1jk}=0,\;\sum_{r=1}^{m_{2}}p_{2r}\delta_{2rs}=0,\\ \sum_{j=1}^{m_{1}}p_{1j}{\left(\alpha \alpha \right)}_{jr}=0,\;\sum_{r=1}^{m_{2}}p_{2r}{\left(\alpha \alpha \right)}_{jr}=0,\;\sum_{j=1}^{m_{1}}p_{1j}{\left(\alpha \delta \right)}_{j,rs}=0,\;\\ \sum_{r=1}^{m_{2}}p_{2r}{\left(\alpha \delta \right)}_{j,rs}=0,\sum_{j=1}^{m_{1}}p_{1j}{\left(\delta \alpha \right)}_{jk,r}=0,\;\sum_{r=1}^{m_{2}}p_{2r}{\left(\delta \alpha \right)}_{jk,r}=0,\\ \;\sum_{j=1}^{m_{1}}p_{1j}{\left(\delta \delta \right)}_{jk,rs}=0,\sum_{r=1}^{m_{2}}p_{2r}{\left(\delta \delta \right)}_{jk,rs}=0 \end{array}$$

In other words, a weighted sum of the average genetic effects of a genetic component is zero over any index. It has been known that model (7) under constraints (8) can provide an orthogonal partition on the genetic variance components when the inheritance of four paternal and maternal alleles at the two loci are independent (Kempthorne, 1957; Weir and Cockerham, 1977). Still, as pointed out in Wang (2014), it is difficult to estimate the parameters in model (7) under the complicated constraints (8) when other adjusted covariates are involved. Besides, the random variables that constitute the random sources of the genetic variance components are not explicitly defined in model (7).

To model the expected genotypic values through a GLM, we can re-list all the sampled individuals by an index variable *k* = 1, … , *N*. Similar to the one-locus case, we introduce the indicator variables $z_{1j}^{\left(1\right)}$ and $z_{2k}^{\left(1\right)}$ for inheritance of the paternal and maternal alleles at locus 1, and $z_{1r}^{\left(2\right)}$ and $z_{2s}^{\left(2\right)}$ for inheritance of the paternal and maternal alleles at locus 2. Then, for phase-unknown genotypes, we define the genotype coding variables *w*_1*j*_, *v*_1*jk*_ for *j, k* = 1, … , *m*_1_ (*j* ≤ *k*) at locus 1, and *w*_2*r*_, *v*_2*rs*_ for *r, s* = 1, … , *m*_2_ (*r* ≤ *s*) at locus 2, in the same way as we did in the one-locus case. By including the within locus additive and dominance effects as well as the locus-by-locus interactions (i.e., epistases), a fully parameterized two-locus GLM model can be written as (Wang, 2011),

(9) $$\begin{array}{l} \text{E}(G|g_{i})=\mu_{0}+\sum_{j=1}^{m_{1}-1}a_{1j}w_{1j}+\sum_{j=1}^{m_{1}-1}\sum_{k=j}^{m_{1}-1}d_{1jk}v_{1jk}+\sum_{r=1}^{m_{2}-1}a_{2r}w_{2r}\\ \;+\sum_{r=1}^{m_{2}-1}\sum_{s=r}^{m_{2}-1}d_{2rs}v_{2rs}+\sum_{j=1}^{m_{1}-1}\sum_{r=1}^{m_{2}-1}{\left(aa\right)}_{jr}w_{1j}w_{2r}\\ \;+\sum_{j=1}^{m_{1}-1}\sum_{r=1}^{m_{2}-1}\sum_{s=r}^{m_{2}-1}{\left(ad\right)}_{j,rs}w_{1j}v_{2rs}\\ \;+\sum_{j=1}^{m_{1}-1}\sum_{k=j}^{m_{1}-1}\sum_{r=1}^{m_{2}-1}{\left(da\right)}_{jk,r}v_{1jk}w_{2r}\\ \;+\sum_{j=1}^{m_{1}-1}\sum_{k=j}^{m_{1}-1}\sum_{r=1}^{m_{2}-1}\sum_{s=r}^{m_{2}-1}{\left(dd\right)}_{jk,rs}v_{1jk}v_{2rs} \end{array}$$

for *i* = 1, …, *N*. A nice feature of the above GLM is that we can easily establish the relationship between its model parameters and the expected genotypic values by starting from the lowest order parameter μ_0_ = *G*_*m*_1_*m*_1_*m*_2_*m*_2__. Suppose that there are no empty genotypes; i.e., *n*_*jkrs*_ > 0 for any *j, k* = 1, … , *m*_1_ and *r, s* = 1, … , *m*_2_. When we incorporate the above model (9) into a GLM (1) and ignore the adjusted covariates, the LSE of parameters in model (9) can be derived as shown in Appendix C in Supplementary Material. Similar to the one-locus GLM, the LSE of these fixed allelic effects are highly dependent upon the genotypic group means, which could be sensitive to phenotypic outliers in small genotypic groups. More importantly, model (9) cannot provide the same partition of the expected genotypic variance *V*(E(*G*|*g*)) as the one defined in the original Fisher's ANOVA model (7).

To construct a two-locus GMA model for analysis of genetic variance components, we can introduce the mean-corrected index variables $x_{1j}^{\left(1\right)},x_{2k}^{\left(1\right)}$ for *j, k* = 1, … , *m*_1_ at locus 1, and $x_{1r}^{\left(2\right)},x_{2s}^{\left(2\right)}$ for *r, s* = 1, … , *m*_2_ at locus 2. Then we define the modified genotype coding variables $w_{1j}^{*}$, $v_{1jk}^{*}$ for *j, k* = 1, … , *m*_1_ at locus 1, and $w_{2r}^{*}$, $v_{2rs}^{*}$ for *r, s* = 1, … , *m*_2_ at locus 2 in the same way as we did in the one-locus case. By including the within locus additive and dominance effects as well as the locus-by-locus interactions (i.e., epistases), we can build a fully parameterized two-locus GMA model as the following.

(10) $$\begin{array}{l} \text{E}(G|g_{i})=\mu^{*}+\sum_{j=1}^{m_{1}-1}\alpha_{1j}^{*}w_{1j}^{*}+\sum_{j=1}^{m_{1}-1}\sum_{k=j}^{m_{1}-1}\delta_{1jk}^{*}v_{1jk}^{*}+\sum_{r=1}^{m_{2}-1}\alpha_{2r}^{*}w_{2r}^{*}\\ \;+\sum_{r=1}^{m_{2}-1}\sum_{s=r}^{m_{2}-1}\delta_{2rs}^{*}v_{2rs}^{*}+\sum_{j=1}^{m_{1}-1}\sum_{r=1}^{m_{2}-1}{\left(\alpha \alpha \right)}_{jr}^{*}w_{1j}^{*}w_{2r}^{*}\\ \;+\sum_{j=1}^{m_{1}-1}\sum_{r=1}^{m_{2}-1}\sum_{s=r}^{m_{2}-1}{\left(\alpha \delta \right)}_{j,rs}^{*}w_{1j}^{*}v_{2rs}^{*}\\ \;+\sum_{j=1}^{m_{1}-1}\sum_{k=j}^{m_{1}-1}\sum_{r=1}^{m_{2}-1}{\left(\delta \alpha \right)}_{jk,r}^{*}v_{1jk}^{*}w_{2r}^{*}\\ \;+\sum_{j=1}^{m_{1}-1}\sum_{k=j}^{m_{1}-1}\sum_{r=1}^{m_{2}-1}\sum_{s=r}^{m_{2}-1}{\left(\delta \delta \right)}_{jk,rs}^{*}v_{1jk}^{*}v_{2rs}^{*} \end{array}$$

for *i* = 1, …, *N*. Similar to the one-locus case, the original Fisher's ANOVA model (7) under constraints (8) and the symmetric properties of the dominance effects can be re-written as

(11) $$\begin{array}{l} \text{E}(G|g_{i})=\mu +\sum_{j=1}^{m_{1}}\alpha_{1j}w_{1j}^{*}+\sum_{j=1}^{m_{1}}\sum_{k=j}^{m_{1}}\delta_{1jk}v_{1jk}^{*}+\sum_{r=1}^{m_{2}}\alpha_{2r}w_{2r}^{*}\\ \;+\sum_{r=1}^{m_{2}}\sum_{s=r}^{m_{2}}\delta_{2rs}v_{2rs}^{*}+\sum_{j=1}^{m_{1}}\sum_{r=1}^{m_{2}}{\left(\alpha \alpha \right)}_{jr}w_{1j}^{*}w_{2r}^{*}\\ \;+\sum_{j=1}^{m_{1}}\sum_{r=1}^{m_{2}}\sum_{s=r}^{m_{2}}{\left(\alpha \delta \right)}_{j,rs}w_{1j}^{*}v_{2rs}^{*}\\ \;+\sum_{j=1}^{m_{1}}\sum_{k=j}^{m_{1}}\sum_{r=1}^{m_{2}}{\left(\delta \alpha \right)}_{jk,r}v_{1jk}^{*}w_{2r}^{*}\\ \;+\sum_{j=1}^{m_{1}}\sum_{k=j}^{m_{1}}\sum_{r=1}^{m_{2}}\sum_{s=r}^{m_{2}}{\left(\delta \delta \right)}_{jk,rs}v_{1jk}^{*}v_{2rs}^{*}. \end{array}$$

Model (10) is actually a simplified version of model (11) by further removing the redundant parameters. The two models (10) and (11) are equivalent when we take

$$\begin{array}{l} \;\mu^{*}=\mu ,\;\alpha_{1j}^{*}=\alpha_{1j}-\alpha_{1m_{1}},\;\alpha_{2k}^{*}=\alpha_{2k}-\alpha_{2m_{2}}\\ \;\delta_{1jk}^{*}=\delta_{1jk}-\delta_{1jm_{1}}-\delta_{1km_{1}}+\delta_{m_{1}m_{1}}\\ \;\delta_{2rs}^{*}=\delta_{2rs}-\delta_{2rm_{2}}-\delta_{2sm_{2}}+\delta_{m_{2}m_{2}}\\ \;{\left(\alpha \alpha \right)}_{jr}^{*}={\left(\alpha \alpha \right)}_{jr}-{\left(\alpha \alpha \right)}_{jm_{2}}-{\left(\alpha \alpha \right)}_{m_{1}r}+{\left(\alpha \alpha \right)}_{m_{1}m_{2}}\\ \;{\left(\alpha \delta \right)}_{j,rs}^{*}=[{\left(\alpha \delta \right)}_{j,rs}-{\left(\alpha \delta \right)}_{j,rm_{2}}-{\left(\alpha \delta \right)}_{j,sm_{2}}+{\left(\alpha \delta \right)}_{j,m_{2}m_{2}}]\\ \;-[{\left(\alpha \delta \right)}_{m_{1},rs}-{\left(\alpha \delta \right)}_{m_{1},rm_{2}}-{\left(\alpha \delta \right)}_{m_{1},sm_{2}}+{\left(\alpha \delta \right)}_{m_{1},m_{2}m_{2}}]\\ \;{\left(\delta \alpha \right)}_{jk,r}^{*}=[{\left(\delta \alpha \right)}_{jk,r}-{\left(\delta \alpha \right)}_{jm_{1},r}-{\left(\delta \alpha \right)}_{km_{1},r}+{\left(\delta \alpha \right)}_{m_{1}m_{1},r}]\\ \;-[{\left(\delta \alpha \right)}_{jk,m_{2}}-{\left(\delta \alpha \right)}_{jm_{1},m_{2}}-{\left(\delta \alpha \right)}_{km_{1},m_{2}}+{\left(\delta \alpha \right)}_{m_{1}m_{1},m_{2}}]\\ {\left(\delta \delta \right)}_{jk,rs}^{*}=[{\left(\delta \delta \right)}_{jk,rs}-{\left(\delta \delta \right)}_{jk,rm_{2}}-{\left(\delta \delta \right)}_{jk,sm_{2}}+{\left(\delta \delta \right)}_{jk,m_{2}m_{2}}]\\ \;-[{\left(\delta \delta \right)}_{jm_{1},rs}-{\left(\delta \delta \right)}_{jm_{1},rm_{2}}-{\left(\delta \delta \right)}_{jm_{1},sm_{2}}+{\left(\delta \delta \right)}_{jm_{1},m_{2}m_{2}}]\\ \;-[{\left(\delta \delta \right)}_{km_{1},rs}-{\left(\delta \delta \right)}_{km_{1},rm_{2}}-{\left(\delta \delta \right)}_{km_{1},sm_{2}}+{\left(\delta \delta \right)}_{km_{1},m_{2}m_{2}}]\\ \;+[{\left(\delta \delta \right)}_{m_{1}m_{1},rs}-{\left(\delta \delta \right)}_{m_{1}m_{1},rm_{2}}-{\left(\delta \delta \right)}_{m_{1}m_{1},sm_{2}}\\ \;+{\left(\delta \delta \right)}_{m_{1}m_{1},m_{2}m_{2}}] \end{array}$$

for *j, k* = 1, … , *m*_1_ − 1 (*j* ≤ *k*) and *r, s* = 1, … , *m*_2_ − 1 (*r* ≤ *s*). Therefore, both model (10) and (11) share exactly the same genetic components as the ones defined in the original Fisher's two-locus ANOVA model (7). In model (10), let $A_{1}=\overset{m_{1}-1}{\underset{j=1}{\sum }}\alpha_{1j}^{*}w_{1j}^{*}$, $D_{1}=\overset{m_{1}-1}{\underset{j=1}{\sum }}\overset{m_{1}-1}{\underset{k=j}{\sum }}\delta_{1jk}^{*}v_{1jk}^{*}$, $A_{2}=\overset{m_{2}-1}{\underset{r=1}{\sum }}\alpha_{2r}^{*}w_{2r}^{*}$, $D_{2}=\overset{m_{2}-1}{\underset{r=1}{\sum }}\overset{m_{2}-1}{\underset{s=r}{\sum }}\delta_{2rs}^{*}v_{2rs}^{*}$, $A_{1}A_{2}=\overset{m_{1}-1}{\underset{j=1}{\sum }}\overset{m_{2}-1}{\underset{r=1}{\sum }}{\left(\alpha \alpha \right)}_{jr}^{*}w_{1j}^{*}w_{2r}^{*}$, $A_{1}D_{2}=\overset{m_{1}-1}{\underset{j=1}{\sum }}\overset{m_{2}-1}{\underset{r=1}{\sum }}\overset{m_{2}-1}{\underset{s=r}{\sum }}{\left(\alpha \delta \right)}_{j,rs}^{*}w_{1j}^{*}v_{2rs}^{*}$, $D_{1}A_{2}=\overset{m_{1}-1}{\underset{j=1}{\sum }}\overset{m_{1}-1}{\underset{k=j}{\sum }}\overset{m_{2}-1}{\underset{r=1}{\sum }}{\left(\delta \alpha \right)}_{jk,r}^{*}v_{1jk}^{*}w_{2r}^{*}$ and $D_{1}D_{2}=\overset{m_{1}-1}{\underset{j=1}{\sum }}\overset{m_{1}-1}{\underset{k=j}{\sum }}\overset{m_{2}-1}{\underset{r=1}{\sum }}\overset{m_{2}-1}{\underset{s=r}{\sum }}{\left(\delta \delta \right)}_{jk,rs}^{*}v_{1jk}^{*}v_{2rs}^{*}$, which represent the additive and dominance at locus 1, additive and dominance at locus 2, additive by additive, additive by dominance, dominance by additive and dominance by dominance genetic components, respectively. Each genetic component consists of a set of average allelic effects (additive or interaction) contributed by alleles from the same locus or the same set of loci, and at each locus at most two alleles could be involved. We refer the number of alleles involved in each average allelic effect of a genetic component as the order of the genetic component, and the number of non-redundant average allelic effects involved in a genetic component as the degrees of freedom (*df*) of the genetic component. Note that genetic components of the same order may have different *df*. For example, the additive by additive component *A*_1_*A*_2_ has its order being 2 and *df* = (*m*_1_ − 1)(*m*_2_ − 1), while the dominance component *D*_1_ at locus 1 has its order being 2 and $df={\left(m_{1}-1\right)}^{2}$.

Both the GLM (9) and GMA model (10) provide a re-parameterization of the expected genotypic values. From the relationship between their genotype coding variables, these two models can be transformed equivalently from one to the other. We can represent the parameters in GMA model (10) in terms of the parameters in its equivalent GLM (9) as shown in Appendix D in Supplementary Material. It is interesting to see that an average allelic effect can be affected by not only its corresponding fixed allelic effect but also other higher-order fixed allelic effects. As a result, the hypothesis test of an average allelic effect in the GMA model (10) is not equivalent to the hypothesis test of the corresponding fixed allelic effect in an equivalent GLM (9) when higher order fixed allelic effects are present. From Appendices C, D in Supplementary Material, we can also derive the LSE of parameters for the GMA model (10) as the following

$$\begin{array}{l} \;{\hat{\mu }}^{*}={\bar{y}}_{\cdot \cdot \cdot \cdot }^{*},\;{\hat{\alpha }}_{1j}^{*}={\bar{y}}_{j\cdot \cdot \cdot }^{*}-{\bar{y}}_{m_{1}\cdot \cdot \cdot }^{*},\;{\hat{\alpha }}_{2r}^{*}={\bar{y}}_{\cdot \cdot r\cdot }^{*}-{\bar{y}}_{\cdot \cdot m_{2}\cdot }^{*}\\ \;{\hat{\delta }}_{1jk}^{*}={\bar{y}}_{jk\cdot \cdot }^{*}-({\bar{y}}_{jm_{1}\cdot \cdot }^{*}+{\bar{y}}_{m_{1}k\cdot \cdot }^{*})+{\bar{y}}_{m_{1}m_{1}\cdot \cdot }^{*}\\ \;{\hat{\delta }}_{2rs}^{*}={\bar{y}}_{\cdot \cdot rs}^{*}-({\bar{y}}_{\cdot \cdot rm_{2}}^{*}+{\bar{y}}_{\cdot \cdot m_{2}s}^{*})+{\bar{y}}_{\cdot \cdot m_{2}m_{2}}^{*}\\ \;{\hat{\left(\alpha \alpha \right)}}_{jr}^{*}={\bar{y}}_{j\cdot r\cdot }^{*}-({\bar{y}}_{j\cdot m_{2}\cdot }^{*}+{\bar{y}}_{m_{1}\cdot r\cdot }^{*})+{\bar{y}}_{m_{1}\cdot m_{2}\cdot }^{*}\\ {\hat{\left(\alpha \delta \right)}}_{j,rs}^{*}={\bar{y}}_{j\cdot rs}^{*}-({\bar{y}}_{m_{1}\cdot rs}^{*}+{\bar{y}}_{j\cdot rm_{2}}^{*}+{\bar{y}}_{j\cdot m_{2}s}^{*})\\ \;+({\bar{y}}_{j\cdot m_{2}m_{2}}^{*}+{\bar{y}}_{m_{1}\cdot rm_{2}}^{*}+{\bar{y}}_{m_{1}\cdot m_{2}s}^{*})-{\bar{y}}_{m_{1}\cdot m_{2}m_{2}}^{*}\\ {\hat{\left(\delta \alpha \right)}}_{jk,r}^{*}={\bar{y}}_{jkr\cdot }^{*}-({\bar{y}}_{jkm_{2}\cdot }^{*}+{\bar{y}}_{jm_{1}r\cdot }^{*}+{\bar{y}}_{km_{1}r\cdot }^{*})\\ \;+({\bar{y}}_{jm_{1}m_{2}\cdot }^{*}+{\bar{y}}_{km_{1}m_{2}\cdot }^{*}+{\bar{y}}_{m_{1}m_{1}r\cdot })-{\bar{y}}_{m_{1}m_{1}m_{2}\cdot }^{*} \end{array}$$

and ${\hat{\left(\delta \delta \right)}}_{jk,rs}^{*}={\hat{\left(dd\right)}}_{jk,rs}$, for *j, k* = 1, …, *m*_1_ − 1, *j* ≤ *k*; and *r, s* = 1, …, *m*_2_ − 1, *r* ≤ *s*. Similar to the one-locus GMA model, the LSE of these average allelic effects depend on the weighted genotypic group means. Except the LSE of the highest order effects for dominance by dominance interactions, the LSE of other lower order average allelic effects could be more robust to phenotypic outliers in small genotypic groups than the LSE of their corresponding fixed allelic effects.

When the two markers are unlinked and in HWE, the inheritance of four paternal and maternal alleles at the two loci are independent. Therefore, the four sets of indicator variables $\left\{z_{1j}^{\left(1\right)},j=1,\ldots \;,m_{1}\right\}$, $\left\{z_{2k}^{\left(1\right)},k=1,\ldots \;,m_{1}\right\}$, $\left\{z_{1r}^{\left(2\right)},r=1,\ldots \;,m_{2}\right\}$, and $\left\{z_{2s}^{\left(2\right)},s=1,\ldots \;,m_{2}\right\}$ are independent with each other, although the variables within each set could still be correlated. In this case, all the genetic components are independent of each other, and the GMA model (10) can provide an orthogonal partition of the expected genotypic variance. In Wang (2014), we have derived formulas for computing the genetic variance components based on the model parameters in a general multi-locus GMA model. For the two-locus GMA model (10), by plugging in the LSE of its model parameters, we obtain estimators of the genetic variance components as shown in Appendix E in Supplementary Material. It should be pointed out that Weir and Cockerham (1977) also derived estimates of the genetic variance components based on the model parameters in Fisher's two-locus ANOVA model (7). But they did not construct the estimators of genetic variance components in terms of the weighted genotypic group means. Note that the orthogonal partition on the genetic variance components implies that the genetic components constitute independent random sources contributing to the expected genotypic variance. Therefore, we could estimate and test for each genetic component separately. Bonferroni criterion can also be applied to correct for the association testing of multiple genetic components.

When the inheritance of paternal and maternal alleles at the two loci are dependent, non-zero covariances among different genetic components may present. The dependency among inheritance of the paternal and maternal alleles at the two markers can be measured by *D*_*jkrs*_ = *p*_*jkrs*_/(2 − 1_{*j* = *k*}_)(2 − 1_{*r* = *s*}_)−*p*_*j*_*p*_*k*_*p*_*r*_*p*_*s*_, for *j, k* = 1, … , *m*_1_ and *r, s* = 1, … , *m*_2_. Let ${\hat{D}}_{jkrs}={\hat{p}}_{jkrs}/\left(2-1_{\left\{j=k\right\}}\right)\left(2-1_{\left\{r=s\right\}}\right)-{\hat{p}}_{j}{\hat{p}}_{k}{\hat{p}}_{r}{\hat{p}}_{s}$. Similar to the one-locus case, when ${\hat{D}}_{jkrs}=0$ for *j, k* = 1, … , *m*_1_ and *r, s* = 1, … , *m*_2_, we can show that the LSE of the average additive, dominance, additive by additive, additive by dominance, dominance by additive and dominance by dominance effects in the full model (10) can keep consistent when some components are excluded from the model.

In general, we can always incorporate the two-locus GMA model (10) into a regression model (1). By treating the modified genotype coding variables $w_{1j}^{*}$, $w_{2r}^{*}$, $v_{1jk}^{*}$, and $v_{2rs}^{*}$ as regular fixed covariates, we can fit the regression model using the ordinary LS approach. Based on the fitted model, we can calculate the fitted genetic components for each individual *i* = 1, … , *N*. Then the genetic variance components and their possible covariances can be estimated as the sample variances and covariances from the fitted values of these genetic components. Similar to the one-locus case, these estimators of the variance and covariance components are different from the traditional ANOVA estimators when the two marker are linked or their genotypes are in HWD. As the estimators of variance components coming from the sample variances, they are guaranteed to be non-negative. When the model residuals are normally distributed, these estimators of the variance and covariance components become MLE and likely possess the asymptotic normality property. Besides, these variance and covariance estimators are likely asymptotically unbiased.

### 2.3. A simulation example

Consider two biallelic marker loci with allele frequencies *p*_1_ = 0.4 and *q*_1_ = 0.6 for alleles “1” and “0” at locus 1, and *p*_2_ = 0.2 and *q*_2_ = 0.8 for alleles “1” and “0” at locus 2. Assume that the two marker loci are in linkage and gametic equilibria. The expected genotypic values at the two marker loci are simulated based on a two-locus GLM model (9) with μ_0_ = 10 and *a*_11_ = *a*_21_ = *d*_111_ = *d*_211_ = (*aa*)_11_ = 1 and (*ad*)_1, 11_ = (*da*)_11, 1_ = (*dd*)_11, 11_ = 0. From Appendix D in Supplementary Material, we can show that this GLM is equivalent to a GMA model (10) with μ^*^ = 11.72 and $\alpha_{11}^{*}=1.8$, $\alpha_{21}^{*}=2$, $\delta_{111}^{*}=1$, $\delta_{211}^{*}=1$, ${\left(\alpha \alpha \right)}_{11}^{*}=1$, ${\left(\alpha \delta \right)}_{1,11}^{*}=0$, ${\left(\delta \alpha \right)}_{11,1}^{*}=0$, and ${\left(\delta \delta \right)}_{11,11}^{*}=0$. Based on the true expected genotypic values and the genotypic distribution, we also know that the total expected genotypic variance, which is *V*_*G*_ = 3.09, has an orthogonal partition which consists of eight variance components contributed by $w_{1}^{*},v_{1}^{*},w_{2}^{*},v_{2}^{*},ww^{*},wv^{*},vw^{*},vv^{*}$. Besides, these eight components $w_{1}^{*},v_{1}^{*},w_{2}^{*},v_{2}^{*},ww^{*},wv^{*},vw^{*},vv^{*}$ contribute 50.74, 1.87, 41.53, 0.83, 5.03, 0.00, 0.00, 0.00% of the total expected genotypic variance *V*_*G*_. For each random sample of size n, we first generate genotypes of individuals independently based on the genotypic distribution. Then, based on the genotypes, we create dummy variables *w*_1_ = *w*_11_, *v*_1_ = *v*_111_, *w*_2_ = *w*_21_, *v*_2_ = *v*_211_, *ww* = *w*_11_*w_21_, *wv* = *w*_11_*v_211_, *vw* = *v*_111_*w_21_, *vv* = *v*_111_*v_211_ and mean-corrected index variables $w_{1}^{*}=w_{11},v_{1}^{*}=v_{111},w_{2}^{*}=w_{21},v_{2}^{*}=v_{211},ww^{*}=w_{11}*w_{21},wv^{*}=w_{11}*v_{211},vw^{*}=v_{111}*w_{21},vv^{*}=v_{111}*v_{211}$. The phenotypic values is a sum of the genotypic values and the residuals with the residuals being independent of the genotypes. To generate phenotypic values, we assume that the residuals ϵ_*i*_, *i* = 1, … , *n*, are i.i.d. and normally distributed with the residual variance being *V*_ϵ_ = 17.51, which account for 85% of the total phenotypic variance (i.e., the broad sense heritability is about 15%).

First, we show that the GLM and GMA models provide different partitions of the expected genotypic variance. To minimize the sampling variation, we simulate a random sample with *n* = 100,000. We fit both GLM and GMA models to the random sample using SAS “proc glm” (SAS Institute, INC.). For the fitted GLM, we have LSE ${\hat{\mu }}_{0}=9.99$, â_11_ = 1.01, â_21_ = 1.05, ${\hat{d}}_{111}=0.97$, ${\hat{d}}_{211}=0.95$, ${\hat{\left(aa\right)}}_{11}=0.97$, ${\hat{\left(ad\right)}}_{1,11}=0.02$, ${\hat{\left(da\right)}}_{11,1}=-0.03$ and ${\hat{\left(dd\right)}}_{11,11}=0.07$, which are close to the true values. From the fitted GLM, we calculate estimates of the variance components contributed by *w*_1_, *v*_1_, *w*_2_, *v*_2_, *ww, wv, vw, vv* as 0.4894, 0.1274, 0.3533, 0.0345, 0.4153, 0.00, 0.00, 0.00, respectively. The summation of these variance components is 1.4199, which is about 46% of the total expected genotypic variance estimate *V*_*G*_ = 3.0896. In other words, in the GLM based partition of the expected genotypic variance, about 54% is contributed by the covariance components due to the collinearity among the variables *w*_1_, *v*_1_, *w*_2_, *v*_2_, *ww, wv, vw, vv*. For the fitted GMA, we have LSE ${\hat{\mu }}^{*}=11.72$, ${\hat{\alpha }}_{11}^{*}=1.78$, ${\hat{\alpha }}_{21}^{*}=2.02$, ${\hat{\delta }}_{111}^{*}=0.96$, ${\hat{\delta }}_{211}^{*}=0.98$, ${\hat{\left(\alpha \alpha \right)}}_{11}^{*}=0.97$, ${\hat{\left(\alpha \delta \right)}}_{1,11}^{*}=0.05$, ${\hat{\left(\delta \alpha \right)}}_{11,1}^{*}=-0.02$ and ${\hat{\left(\delta \delta \right)}}_{11,11}^{*}=0.07$, which are also close to the true values. From the fitted GMA model, we compute estimates of the variance components contributed by $w_{1}^{*},v_{1}^{*},w_{2}^{*},v_{2}^{*},ww^{*},wv^{*}$, vw^*^, vv^*^ as 1.5321, 0.0534, 1.3079, 0.0244, 0.1462, 0.00, 0.00, 0.00, respectively. The summation of these variance components is 3.064, which is about 99% of the total expected genotypic variance estimate *V*_*G*_ = 3.0896. This indicates that the GMA model leads to a partition of the expected genotypic variance, which is almost orthogonal with only about 1% coming from the covariance components. Note also that these estimates of the variance components contributed by $w_{1}^{*},v_{1}^{*},w_{2}^{*},v_{2}^{*},ww^{*},wv^{*}$, vw^*^, vv^*^ account for approximately 49.59, 1.73, 42.33, 0.79, 4.73, 0.00, 0.00, 0.00% of the expected genotypic variance estimate *V*_*G*_ = 3.0896, which are close to the true proportions 50.74, 1.87, 41.53, 0.83, 5.03, 0.00, 0.00, 0.00% of the variables $w_{1}^{*},v_{1}^{*},w_{2}^{*},v_{2}^{*},ww^{*},wv^{*}$, vw^*^, vv^*^ contributed to the expected genotypic variance.

We also look at the difference between the partition of the expected genotypic variance and the Type III sums of squares for this random sample. Both the GLM and GMA models have the same regression sum of squares *SSR* = 307788.89 and mean square error *MSE* = 17.52. In the fitted GLM, the Type III sums of squares for the eight variables *w*_1_, *v*_1_, *w*_2_, *v*_2_, *ww, wv, vw, vv* are 13415.25, 3475.46, 8472.60, 840.54, 4149.25, 0.24, 1.04, and 0.67, respectively. The summation of these Type III sums of squares is 30355.05, which is less than 10% of the total *SSR*. In the fitted GMA, the Type III sums of squares for the eight variables $w_{1}^{*},v_{1}^{*},w_{2}^{*},v_{2}^{*},ww^{*},wv^{*},vw^{*},vv^{*}$ are 153184.54, 5340.61, 130751.17, 2438.75, 14620.38, 2.92, 0.44, and 0.67, respectively. The summation of these Type III sums of squares is 306339.50, which is about 99.5% of the total *SSR*. This indicates that these Type III sums of squares also provide an orthogonal partition of the *SSR*. Or, in other words, the hypothesis tests of the eight genetic components are approximately orthogonal.

Next, we examine the performance in model selection between using the GLM and GMA models. We consider varied sample sizes of *n* = 500, 1000, 2000, and 5000. Under each simulation scenario, we run 1000 simulation. For each simulation sample, we first apply one commonly used method—the forward stepwise selection for model selection on {*w*_1_, *v*_1_, *w*_2_, *v*_2_, *ww, wv, vw, vv*} and $\left\{w_{1}^{*},v_{1}^{*},w_{2}^{*},v_{2}^{*},ww^{*},wv^{*},vw^{*},vv^{*}\right\}$, separately. We run “proc glmselect” in SAS with the criterion p=0.05 for both entry and stay in the model. For the GMA model selection on index variables $\left\{w_{1}^{*},v_{1}^{*},w_{2}^{*},v_{2}^{*},ww^{*},wv^{*},vw^{*},vv^{*}\right\}$, as these variables are independent in the underlying true model, we can rank them in the order of $w_{1}^{*},w_{2}^{*},ww^{*},v_{1}^{*},v_{2}^{*},vw^{*},wv^{*}$, and vv^*^ according to their variance contributions from the largest to the smallest. Intuitively, we expect that the selected models would include the higher ranked variables more often than the lower ranked ones. We mainly focus on the top five variables $w_{1}^{*},w_{2}^{*},ww^{*},v_{1}^{*},v_{2}^{*}$ and classify the selected models into 10 categories: I = all the five variables $w_{1}^{*},w_{2}^{*},ww^{*},v_{1}^{*},v_{2}^{*}$ are selected; II = $w_{1}^{*},w_{2}^{*},ww^{*},v_{1}^{*}$ are selected but not $v_{2}^{*}$; III = $w_{1}^{*},w_{2}^{*},ww^{*},v_{2}^{*}$ are selected but not $v_{1}^{*}$; IV= $w_{1}^{*},w_{2}^{*},ww^{*}$ are selected but not $v_{1}^{*},v_{2}^{*}$; V= $w_{1}^{*},w_{2}^{*}$ are selected but not $ww^{*},v_{1}^{*},v_{2}^{*}$; VI = $w_{1}^{*},ww^{*}$ are selected but not $w_{2}^{*}$; VII = $w_{2}^{*},ww^{*}$ are selected but not $w_{1}^{*}$; VIII = ww^*^ is selected but $w_{1}^{*},w_{2}^{*}$ are missed; IX=$w_{1}^{*}$ is selected but miss $w_{2}^{*},ww^{*}$, or $w_{2}^{*}$ is selected but miss $w_{1}^{*},ww^{*}$; X=$w_{1}^{*},w_{2}^{*},ww^{*}$ are missed. For the GLM model selection on the dummy variables {*w*_1_, *v*_1_, *w*_2_, *v*_2_, *ww, wv, vw, vv*}, we also define the similar 10 categories I-VIII based on the variables *w*_1_, *v*_1_, *w*_2_, *v*_2_, *ww, wv, vw, vv*. Under each simulation scenario, the counts of selected GLM and GMA model types for stepwise selection are listed in Table 1.

**Table 1:** **Counts of selected GLM and GMA model types for “Stepwise Selection”**.

**Model types**	**GLM**				**GMA**			
	***n* = 500**	***n* = 1000**	***n* = 2000**	***n* = 5000**	***n* = 500**	***n* = 1000**	***n* = 2000**	***n* = 5000**
I	0	15	196	605	13	71	285	737
II	3	132	401	342	118	294	433	239
III	0	27	70	33	43	98	99	22
IV	89	266	176	9	370	361	160	2
V	288	213	71	11	456	176	23	0
VI	247	140	47	0	0	0	0	0
VII	82	77	9	0	0	0	0	0
VIII	265	129	30	0	0	0	0	0
IX	26	1	0	0	0	0	0	0
X	0	0	0	0	0	0	0	0
Total	1000	1000	1000	1000	1000	1000	1000	1000

For the stepwise selection, the GMA model shows a clear advantage over the GLM on choosing the Type I (the true model), Type I+II, Type I+II+III, Type I+II+III+IV, or Type I-V models. Meanwhile, the GLM tends to miss one of the main fixed effects (Type VI, VII, or VIII) when the sample size ≤ 2000, even though a Type VI, VII, or VIII GLM could imply an equivalent GMA model of Type IV. Overall, as the sample size increases, the model selection improves using either GLM or GMA models. But the selected GLM have a bigger variety, while the selected GMA models are more predictable.

It has been known that the conventional stepwise model selection has problems such as the stepwise F-statistics may no longer follow the F-distributions and the aggravated collinearity among the selected variables (Harrell, 2001). Several regularized regression methods have been developed to deal with the model selection problem especially for high-dimensional data. Here we also apply one of the regularized regression methods - the adaptive LASSO, using “proc glmselect” in SAS. We adopt the Bayesian information criterion (BIC) for model selections. The standardization of the variables is also made prior to the model selection. Note that the standardization of the variables {*w*_1_, *v*_1_, *w*_2_, *v*_2_, *ww, wv, vw, vv*} or $\left\{w_{1}^{*},v_{1}^{*},w_{2}^{*},v_{2}^{*},ww^{*},wv^{*},vw^{*},vv^{*}\right\}$ can only affect the scales of the regression coefficients but does not change the correlation structure among each set of variables, while making mean-corrections on the indicator variables $\left\{z_{1j}^{\left(1\right)},z_{2k}^{\left(1\right)}\right\}$ and $\left\{z_{1r}^{\left(2\right)},z_{2s}^{\left(2\right)}\right\}$ can lead to different correlation structures between the dummy variables {*w*_1_, *v*_1_, *w*_2_, *v*_2_, *ww, wv, vw, vv*} and the index variables $\left\{w_{1}^{*},v_{1}^{*},w_{2}^{*},v_{2}^{*},ww^{*},wv^{*},vw^{*},vv^{*}\right\}$. The counts of selected GLM and GMA model types for adaptive LASSO are listed in Table 2.

**Table 2:** **Counts of selected GLM and GMA model types for “Adaptive LASSO”**.

**Model types**	**GLM**				**GMA**			
	***n* = 500**	***n* = 1000**	***n* = 2000**	***n* = 5000**	***n* = 500**	***n* = 1000**	***n* = 2000**	***n* = 5000**
I	1	18	91	363	4	17	58	367
II	17	87	246	372	33	89	226	346
III	8	18	41	19	17	20	38	13
IV	87	180	226	153	194	336	371	197
V	271	227	125	25	602	432	237	55
VI	184	212	150	46	0	0	0	0
VII	39	34	13	0	0	0	0	0
VIII	227	145	71	10	0	0	0	0
IX	61	17	6	1	8	0	0	0
X	105	62	31	11	142	106	70	22
Total	1000	1000	1000	1000	1000	1000	1000	1000

For the adaptive LASSO, it appears that the GMA model performs slightly better on choosing Type I (the true model), Type I+II, Type I+II+III, Type I+II+III+IV, or Type I-V models when sample size=500. However, this advantage diminishes for larger sample sizes. Still, the selected GLM appear to have a bigger variety, while the selected GMA models are more predictable. On the other hand, it seems that the selected GMA models could miss all the three top variables $w_{1}^{*},w_{2}^{*},ww^{*}$ more likely than the GLM models for *w*_1_, *w*_2_, *ww*. A brief comparison between Tables 1, 2 also reveals that the stepwise selection has a better performance than the adaptive LASSO under our simulation setting. But this may not be a fair comparison as the selected variables in our simulation setting is very limited. Besides, except BIC, many other criteria are available for model selections in “proc glmselect.” Further exploration on those criteria is needed.

## 3. Discussion

In this study, we applied the GMA models on analysis of variance components for genetic markers with unphased genotypes. We pointed out that the traditional Fisher's ANOVA model does not explicitly specify the random variables that contribute to the various genetic variance components. The constraints on their model parameters also complicate the model fitting. Meanwhile, the classical dummy-variable based GLM does not provide the same partition on the genetic variance components as the original Fisher's ANOVA model. The hypothesis tests of the fixed allelic effects in a GLM based on the partial (Type III) reduction in sums of squares could also be inadequate for testing the existence of variance components when allelic interactions are present. Alternatively, the GMA model can retain the same partition on the genetic variance components as the traditional Fisher's ANOVA model. Similar to the classical GLM, the GMA model does not require constraints on its model parameters and can be fitted using the ordinary least square approach. As the result, the GMA model allows us to estimate and test for the genetic variance components more conveniently than the classical GLM.

The classical GLM is appealing in genetic association studies due to its simplicity in interpretation of the model parameters, which often represent certain comparisons of the expected genotypic values in different genotype groups. However, the GLM-based approach faces challenges in dealing with allelic interactions because the fixed lower order allelic effects are often confounded with the higher order allelic interactions in a GLM even when the paternal and maternal alleles are independently inherited. In order to test for a particular fixed allelic interaction based on the classical GLM, we need to include all its lower order effects in the model to make this allelic interaction interpretable. Besides, ignoring certain higher order interactions in the model could also affect the definition of this particular allelic interaction due to their potential confounding, as pointed out in Zeng et al. (2005). On the other hand, analysis of genetic variance components using ANOVA type models such as GMA provides an alternative way on assessing the allelic effects and interactions. A nice feature of the GMA model is that the genetic components are independent of each other when multiple genetic markers are unlinked and in equilibrium populations. Therefore, at least in equilibrium populations, each genetic component can be treated as a random source of variation and tested individually regardless of its lower or higher order genetic components. A statistically significant higher-order genetic variance component implies a variation contributed by varied allele types from a set of loci, which could be more perceivable than a significant fixed allelic interaction for claiming allelic interactions.

As shown in Wang (2014), the extension of GMA to multiple loci is straightforward. The GMA model could also be applied to other phenotypic outcomes. For example, under the generalized linear mixed model framework, we can consider the genetic components contributing to a g-transformed (g - a link function) expected outcomes. For survival outcomes, we can examine the genetic components contributing to the hazard functions as well. However, it should be pointed out that the estimation of genetic variance components for genetic markers relies on the genotypic distribution of the markers in a study population. When a sample does not come from the simple random sampling, an adjustment for the sampling strategy is needed in order to make appropriate statistical inference in the general population. Currently, the genome-wide association studies (GWAS) often adopt the case-control design. When the case and control sampling rates are known, we could assess the variance components of genetic markers based on their odds ratio estimates. A thorough exploration on applying the GMA type models to a case-control based GWAS could be a research topic in the future.

## Author contributions

TW planned the study, conducted the derivation, and wrote the manuscript.

### Conflict of interest statement

The author declares that the research was conducted in the absence of any commercial or financial relationships that could be construed as a potential conflict of interest. The reviewer ZY and handling editor declared their shared affiliation, and the handling editor states that the process nevertheless met the standards of a fair and objective review.
