# Supplementary material for: Analysis of Variance Components for Genetic Markers with Unphased Genotypes
Source: Front Genet. 2016 Jul 13;7:123. doi: 10.3389/fgene.2016.00123 (PMC4942470; doi:10.3389/fgene.2016.00123)
Supplement: Supplementary file 1 [file DataSheet1.pdf]

Supplementary Material:
Analysis of variance components for genetic markers with unphased genotypes

Tao Wang*
*Correspondence:
Division of Biostatistics, Institute for Health and Society, Medical College of Wisconsin, 8701 Watertown Plank Road, Milwaukee, WI 53226, USA,
taowang@mcw.edu

APPENDIX A. ESTIMATORS OF GENETIC VARIANCE COMPONENTS FOR ONE-LOCUS GMA MODEL (5).

The formulas for estimating the variance components V_A, V_D and the covariance component $\mathrm{Cov}(A, D)$ based on model (5) were provided in Appendix B of Wang (2014). By plugging in the LSE of the parameters in model (5) and the MLE of allele frequencies, we obtain the following estimators of the variance components V_A, V_D and the covariance component $\mathrm{Cov}(A, D)$.

$$\widehat{V}_A = 2\sum_{j=1}^{m} \hat{p}_j(\bar{y}^*_{j\cdot} - \bar{y}^*_{\cdot\cdot})^2 + 2\sum_{j=1}^{m}\sum_{k=1}^{m} D_{jk}\bar{y}^*_{j\cdot}\bar{y}^*_{k\cdot}$$

$$\widehat{V}_D = \sum_{j=1}^{m}\sum_{k=1}^{m} \hat{p}_j\hat{p}_k(\bar{y}_{jk\cdot} - \bar{y}^*_{\cdot\cdot})^2 - 2\sum_{j=1}^{m} \hat{p}_j(\bar{y}^*_{j\cdot} - \bar{y}^*_{\cdot\cdot})^2$$
$$+ \sum_{j=1}^{m}\sum_{k=1}^{m} D_{jk}(\bar{y}_{jk\cdot} - \bar{y}^*_{j\cdot} - \bar{y}^*_{k\cdot} + y^*_{\cdot\cdot})^2 - \Delta_0^2$$

$$\widehat{\mathrm{Cov}(A, D)} = 2\sum_{j=1}^{m}(\bar{y}^*_{j\cdot} - y^*_{\cdot\cdot})\sum_{k=1}^{m} D_{jk}(\bar{y}_{jk\cdot} - \bar{y}^*_{k\cdot})$$

where $\Delta_0 = \sum_{j=1}^m \sum_{k=1}^m D_{jk}\bar{y}_{jk\cdot}$.

APPENDIX B. IN ONE-LOCUS GMA MODEL (5), (X'X) IS A BLOCK DIAGONAL MATRIX WHEN $\hat{D}_{JK} = 0$, FOR $J, K = 1, \cdots, M$.

When $\hat{D}_{jk} = 0$ for $j, k = 1, \cdots, m$, we have $n_{jj}/N = \hat{p}_j^2$ for $j = 1, \cdots, m$, and $n_{jk}/N = 2\hat{p}_j\hat{p}_k$ for $j, k = 1, \cdots, m$ and $j \neq k$. Therefore,

$$\sum_{i=1}^{N} w_j^*(g_i) = \sum_{i=1}^{N}(w_j(g_i) - 2\hat{p}_j) = (2n_{jj} + \sum_{k\neq j} n_{jk}) - 2N\hat{p}_j = 0$$

$$\sum_{i=1}^{N} v_{jj}^*(g_i) = \sum_{i=1}^{N}(v_{jj}(g_i) - \hat{p}_j w_j(g_i) + \hat{p}_j^2) = n_{jj} - \hat{p}_j(2n_{jj} + \sum_{k\neq j} n_{jk}) + N\hat{p}_j^2 = 0$$

for $j = 1, \cdots, m-1$, and

$$\begin{aligned}\sum_{i=1}^{N} v_{jk}^*(g_i) &= \sum_{i=1}^{N}(v_{jk}(g_i) - \hat{p}_j w_k(g_i) - \hat{p}_k w_j(g_i) + 2\hat{p}_j\hat{p}_k) \\ &= n_{jk} - \hat{p}_j(2N\hat{p}_k) - \hat{p}_k(2N\hat{p}_j) + 2N\hat{p}_j\hat{p}_k = 0\end{aligned}$$

for $j, k = 1, \cdots, m$ and $j \neq k$. Thus, $1'_N X_{\alpha^*} = 0_{1\times(m-1)}$ and $1'_N X_{\delta^*} = 0_{1\times\frac{m(m-1)}{2}}$. Moreover, notice that

$$\sum_{i=1}^{N} w_j^*(g_i)v_{j'k}^*(g_i) = \sum_{i=1}^{N}(w_j(g_i) - 2\hat{p}_j)v_{j'k}^*(g_i) = \sum_{i=1}^{N} w_j(g_i)v_{j'k}^*(g_i)$$

for any $j, j', k = 1, \cdots, m-1$. We can show that

$$\begin{aligned}\sum_{i=1}^{N} w_j^*(g_i)v_{jj}^*(g_i) &= \sum_{i=1}^{N} w_j(g_i)(v_{jj}(g_i) - \hat{p}_j w_j(g_i) + \hat{p}_j^2) = 2n_{jj} - \hat{p}_j\sum_{i=1}^{N} w_j^2(g_i) + \hat{p}_j^2(2N\hat{p}_j) \\ &= 2n_{jj} - \hat{p}_j(2N\hat{p}_j + 2n_{jj}) + 2N\hat{p}_j^3 = 0, \text{ for } j = 1, \cdots, m-1, \\ \sum_{i=1}^{N} w_j^*(g_i)v_{kk}^*(g_i) &= \sum_{i=1}^{N} w_j(g_i)(v_{kk}(g_i) - \hat{p}_k w_k(g_i) + \hat{p}_k^2) = -\hat{p}_k n_{jk} + \hat{p}_k^2(2N\hat{p}_j) = 0\end{aligned}$$

for $j, k = 1, \cdots, m-1$, $j \neq k$, and

$$\begin{aligned}\sum_{i=1}^{N} w_{j'}^*(g_i)v_{jk}^*(g_i) &= \sum_{i=1}^{N} w_{j'}(g_i)(v_{jk}(g_i) - \hat{p}_j w_k(g_i) - \hat{p}_k w_j(g_i) + 2\hat{p}_j\hat{p}_k) \\ &= -\hat{p}_j n_{j'k} - \hat{p}_k n_{j'j} + 2\hat{p}_j\hat{p}_k(2N\hat{p}_{j'}) = 0\end{aligned}$$

for $j, j', k = 1, \cdots, m-1$, $j \neq j'$ and $j, j' < k$. In addition, we have

$$\begin{aligned}\sum_{i=1}^{N} w_j^*(g_i)v_{jk}^*(g_i) &= \sum_{i=1}^{N} w_j(g_i)(v_{jk}(g_i) - \hat{p}_j w_k(g_i) - \hat{p}_k w_j(g_i) + 2\hat{p}_j\hat{p}_k) \\ &= \sum_{i=1}^{N} w_j(g_i)v_{jk}(g_i) - \hat{p}_j\sum_{i=1}^{N} w_j(g_i)w_k(g_i) - \hat{p}_k\sum_{i=1}^{N} w_j^2(g_i) + 2\hat{p}_j\hat{p}_k(2N\hat{p}_j) \\ &= n_{jk} - \hat{p}_j n_{jk} - \hat{p}_k(2n_{jj} + 2N\hat{p}_j) + 4N\hat{p}_j^2\hat{p}_k = 0\end{aligned}$$

for $j,k=1,\cdots,m-1$ and $j<k$. Similarly, $\sum_{i=1}^{N} w_k^*(g_i)v_{jk}^*(g_i) =$ for $j,k=1,\cdots,m-1$ and $j<k$. Therefore, $X'_{\alpha^*}X_{\delta^*} = 0_{(m-1)\times\frac{m(m-1)}{2}}$.

APPENDIX C. LSE OF PARAMETERS FOR TWO-LOCUS GLM (9).

$$
\begin{cases}
\widehat{\mu_0} & = \bar{y}_{m_1m_1m_2m_2} \\
\widehat{a}_{1j} & = \bar{y}_{jm_1m_2m_2} - \bar{y}_{m_1m_1m_2m_2} \\
\widehat{a}_{2r} & = \bar{y}_{m_1m_1rm_2} - \bar{y}_{m_1m_1m_2m_2} \\
\widehat{d}_{1jk} & = \bar{y}_{jkm_2m_2} - (\bar{y}_{jm_1m_2m_2} + \bar{y}_{m_1km_2m_2}) + \bar{y}_{m_1m_1m_2m_2} \\
\widehat{d}_{2rs} & = \bar{y}_{m_1m_1rs} - (\bar{y}_{m_1m_1rm_2} + \bar{y}_{m_1m_1sm_2}) + \bar{y}_{m_1m_1m_2m_2} \\
\widehat{(aa)}_{jr} & = \bar{y}_{jm_1rm_2} - (\bar{y}_{jm_1m_2m_2} + \bar{y}_{m_1m_1rm_2}) + \bar{y}_{m_1m_1m_2m_2} \\
\widehat{(ad)}_{j,rs} & = \bar{y}_{jm_1rs} - (\bar{y}_{m_1m_1rs} + \bar{y}_{jm_1rm_2} + \bar{y}_{jm_1sm_2}) \\
 & \quad + (\bar{y}_{jm_1m_2m_2} + \bar{y}_{m_1m_1rm_2} + \bar{y}_{m_1m_1sm_2}) - \bar{y}_{m_1m_1m_2m_2} \\
\widehat{(da)}_{jk,r} & = \bar{y}_{jkrm_2} - (\bar{y}_{jkm_2m_2} + \bar{y}_{jm_1rm_2} + \bar{y}_{km_1rm_2}) \\
 & \quad + (\bar{y}_{jm_1m_2m_2} + \bar{y}_{km_1m_2m_2} + \bar{y}_{m_1m_1rm_2}) - \bar{y}_{m_1m_1m_2m_2} \\
\widehat{(dd)}_{jk,rs} & = \bar{y}_{jkrs} - (\bar{y}_{jm_1rs} + \bar{y}_{km_1rs} + \bar{y}_{jkrm_2} + \bar{y}_{jksm_2}) \\
 & \quad + (\bar{y}_{jkm_2m_2} + \bar{y}_{jm_1rm_2} + \bar{y}_{km_1rm_2} + \bar{y}_{jm_1sm_2} + \bar{y}_{km_1sm_2} + \bar{y}_{m_1m_1rs}) \\
 & \quad - (\bar{y}_{jm_1m_2m_2} + \bar{y}_{km_1m_2m_2} + \bar{y}_{m_1m_1rm_2} + \bar{y}_{m_1m_1sm_2}) + \bar{y}_{m_1m_1m_2m_2}
\end{cases}
$$

for $j,k=1,\ldots,m_1-1$, $j \le k$; and $r,s=1,\ldots,m_2-1$, $r \le s$.

APPENDIX D. RELATIONSHIP OF PARAMETERS BETWEEN TWO EQUIVALENT MODELS (9) AND (10).

We can show that the parameters in model (10) can be represented as the following in terms of the parameters in an equivalent model (9).

$$
\begin{aligned}
\mu^* = \mu_0 &+ 2\sum_{j=1}^{m_1-1} p_{1j}a_{1j} + \sum_{j=1}^{m_1-1}\sum_{k=1}^{m_1-1} p_{1j}p_{1k}d_{1jk} + 2\sum_{r=1}^{m_2-1} p_{2r}a_{2r} + \sum_{r=1}^{m_2-1}\sum_{s=1}^{m_2-1} p_{2r}p_{2s}d_{2rs} \\
&+ 4\sum_{j=1}^{m_1-1}\sum_{r=1}^{m_2-1} p_{1j}p_{2r}(aa)_{jr} + 2\sum_{j=1}^{m_1-1}\sum_{r,s=1}^{m_2-1} p_{1j}p_{2r}p_{2s}(ad)_{j,rs} + 2\sum_{j,k=1}^{m_1-1}\sum_{r=1}^{m_2-1} p_{1j}p_{1k}p_{2r}(da)_{jk,r} \\
&+ \sum_{j,k=1}^{m_1-1}\sum_{r,s=1}^{m_2-1} p_{1j}p_{1k}p_{2r}p_{2s}(dd)_{jk,rs}
\end{aligned}
$$

$$\alpha^*_{1j} = a_{1j} + \sum_{k=1}^{m_1-1} p_{1k}d_{1jk} + 2\sum_{r=1}^{m_2-1} p_{2r}(aa)_{jr} + \sum_{r,s=1}^{m_2-1} p_{2r}p_{2s}(ad)_{j,rs}$$

$$+ 2\sum_{k=1}^{m_1-1}\sum_{r=1}^{m_2-1} p_{1k}p_{2r}(da)_{jk,r} + \sum_{k=1}^{m_1-1}\sum_{r,s=1}^{m_2-1} p_{1k}p_{2r}p_{2s}(dd)_{jk,rs}$$

$$\alpha^*_{2r} = a_{2r} + \sum_{s=1}^{m_2-1} p_{2s}d_{2rs} + 2\sum_{j=1}^{m_1-1} p_{1j}(aa)_{jr} + \sum_{j,k=1}^{m_1-1} p_{1j}p_{1k}(da)_{jk,r}$$

$$+ 2\sum_{j=1}^{m_1-1}\sum_{s=1}^{m_2-1} p_{1j}p_{2s}(ad)_{j,rs} + \sum_{j,k=1}^{m_1-1}\sum_{s=1}^{m_2-1} p_{1j}p_{1k}p_{2s}(dd)_{jk,rs}$$

$$\delta^*_{1jk} = d_{1jk} + 2\sum_{r=1}^{m_2-1} p_{2r}(da)_{jk,r} + \sum_{r,s=1}^{m_2-1} p_{2r}p_{2s}(dd)_{jk,rs}$$

$$\delta^*_{2rs} = d_{2rs} + 2\sum_{j=1}^{m_1-1} p_{1j}(ad)_{j,rs} + \sum_{j,k=1}^{m_1-1} p_{1j}p_{1k}(dd)_{jk,rs}$$

$$(\alpha\alpha)^*_{j,r} = (aa)_{jr} + \sum_{s=1}^{m_2-1} p_{2s}(ad)_{j,rs} + \sum_{k=1}^{m_1-1} p_{1k}(da)_{jk,r} + \sum_{k=1}^{m_1-1}\sum_{s=1}^{m_2-1} p_{1k}p_{2s}(dd)_{jk,rs}$$

$$(\alpha\delta)^*_{j,rs} = (ad)_{j,rs} + \sum_{k=1}^{m_1-1} p_{1k}(dd)_{jk,rs}, \;\; (\delta\alpha)^*_{jk,r} = (da)_{jk,r} + \sum_{s=1}^{m_2-1} p_{2s}(dd)_{jk,rs}$$

and $(\delta\delta)^*_{jk,rs} = (dd)_{jk,rs}$, for $j, k = 1, \ldots, m_1-1$, $j \leq k$; and $r, s = 1, \ldots, m_2-1$, $r \leq s$. Here, we define $d_{1kj} = d_{1jk}$, $d_{2sr} = d_{2rs}$, $(ad)_{j,sr} = (ad)_{j,rs}$, $(da)_{kj,r} = (da)_{jk,r}$ and $(dd)_{kj,rs} = (dd)_{jk,rs} = (dd)_{jk,sr}$, for $j, k = 1, \cdots, m_1$ and $r, s = 1, \cdots, m_2$.

APPENDIX E. ESTIMATORS OF GENETIC VARIANCE COMPONENTS FOR TWO-LOCUS GMA MODEL (10) IN AN EQUILIBRIUM POPULATION

$$\widehat{V}_{A_1} = 2\sum_{j=1}^{m_1}\hat{p}_{1j}(\bar{y}^*_{j\cdots} - \bar{y}^*_{\cdots\cdot})^2, \;\; \widehat{V}_{D_1} = \sum_{j,k=1}^{m_1}\hat{p}_{1j}\hat{p}_{1k}(\bar{y}^*_{jk\cdot\cdot} - \bar{y}^*_{\cdots\cdot})^2 - 2\sum_{j=1}^{m_1}\hat{p}_{1j}(\bar{y}^*_{j\cdots} - \bar{y}^*_{\cdots\cdot})^2$$

$$\widehat{V}_{A_2} = 2\sum_{r=1}^{m_2}\hat{p}_{2r}(\bar{y}^*_{\cdot\cdot r\cdot} - \bar{y}^*_{\cdots\cdot})^2, \;\; \widehat{V}_{D_2} = \sum_{r,s=1}^{m_2}\hat{p}_{2r}\hat{p}_{2s}(\bar{y}^*_{\cdot\cdot rs} - \bar{y}^*_{\cdots\cdot})^2 - 2\sum_{r=1}^{m_2}\hat{p}_{2r}(\bar{y}^*_{\cdot\cdot r\cdot} - \bar{y}^*_{\cdots\cdot})^2$$

$$\widehat{V}_{A_1A_2} = 4\Big[\sum_{j=1}^{m_1}\sum_{r=1}^{m_2}\hat{p}_{1j}\hat{p}_{2r}(\bar{y}^*_{j\cdot r\cdot} - \bar{y}^*_{\cdots\cdot})^2 - \sum_{j=1}^{m_1}\hat{p}_{1j}(\bar{y}^*_{j\cdots} - \bar{y}^*_{\cdots\cdot})^2 - \sum_{r=1}^{m_2}\hat{p}_{2r}(\bar{y}^*_{\cdot\cdot r\cdot} - \bar{y}^*_{\cdots\cdot})^2\Big]$$

$$\widehat{V}_{A_1D_2} = 2\Big[\sum_{j=1}^{m_1}\sum_{r,s=1}^{m_2}\hat{p}_{1j}\hat{p}_{2r}\hat{p}_{2s}(\bar{y}^*_{j\cdot rs} - \bar{y}^*_{....})^2 - \sum_{r,s=1}^{m_2}\hat{p}_{2r}\hat{p}_{2s}(\bar{y}^*_{..rs} - \bar{y}^*_{....})^2$$
$$- 2\sum_{j=1}^{m_1}\sum_{r=1}^{m_2}\hat{p}_{1j}\hat{p}_{2r}(\bar{y}^*_{j\cdot r\cdot} - \bar{y}^*_{....})^2 + \sum_{j=1}^{m_1}\hat{p}_{1j}(\bar{y}^*_{j...} - \bar{y}^*_{....})^2 + 2\sum_{r=1}^{m_2}\hat{p}_{2r}(\bar{y}^*_{..r\cdot} - \bar{y}^*_{....})^2\Big]$$

$$\widehat{V}_{D_1A_2} = 2\Big[\sum_{j,k=1}^{m_1}\sum_{r=1}^{m_2}\hat{p}_{1j}\hat{p}_{1k}\hat{p}_{2r}(\bar{y}^*_{jk\cdot r} - \bar{y}^*_{....})^2 - \sum_{j,k=1}^{m_1}\hat{p}_{1j}\hat{p}_{1k}(\bar{y}^*_{jk\cdot\cdot} - \bar{y}^*_{....})^2$$
$$- 2\sum_{j=1}^{m_1}\sum_{r=1}^{m_2}\hat{p}_{1j}\hat{p}_{2r}(\bar{y}^*_{j\cdot r\cdot} - \bar{y}^*_{....})^2 + \sum_{r=1}^{m_2}\hat{p}_{2r}(\bar{y}^*_{...r} - \bar{y}^*_{....})^2 + 2\sum_{j=1}^{m_1}\hat{p}_{1j}(\bar{y}^*_{j...} - \bar{y}^*_{....})^2\Big]$$

$$\widehat{V}_{D_1D_2} = \sum_{j,k=1}^{m_1}\sum_{r,s=1}^{m_2}\hat{p}_{1j}\hat{p}_{1k}\hat{p}_{2r}\hat{p}_{2s}(\bar{y}_{jkrs\cdot} - \bar{y}^*_{....})^2 - 2\sum_{j=1}^{m_1}\sum_{r,s=1}^{m_2}\hat{p}_{1j}\hat{p}_{2r}\hat{p}_{2s}(\bar{y}^*_{j\cdot rs} - \bar{y}^*_{....})^2$$
$$- 2\sum_{j,k=1}^{m_1}\sum_{r=1}^{m_2}\hat{p}_{1j}\hat{p}_{1k}\hat{p}_{2r}(\bar{y}^*_{jk\cdot r} - \bar{y}^*_{....})^2 + \sum_{j,k=1}^{m_1}\hat{p}_{1j}\hat{p}_{1k}(\bar{y}^*_{jk\cdot\cdot} - \bar{y}^*_{....})^2 + \sum_{r,s=1}^{m_2}\hat{p}_{2r}\hat{p}_{2s}(\bar{y}^*_{..rs} - \bar{y}^*_{....})^2$$
$$+ 4\sum_{j=1}^{m_1}\sum_{r=1}^{m_2}\hat{p}_{1j}\hat{p}_{2r}(\bar{y}^*_{j\cdot r\cdot} - \bar{y}^*_{....})^2 - 2\sum_{j=1}^{m_1}\hat{p}_{1j}(\bar{y}^*_{j...} - \bar{y}^*_{....})^2 - 2\sum_{r=1}^{m_2}\hat{p}_{2r}(\bar{y}^*_{...r} - \bar{y}^*_{....})^2$$

Meanwhile, the estimator of the expected genotypic variance is given by

$$\widehat{V}(\mathrm{E}(G|g)) = \sum_{j,k=1}^{m_1}\sum_{r,s=1}^{m_2}\hat{p}_{1j}\hat{p}_{1k}\hat{p}_{2r}\hat{p}_{2s}(\bar{y}_{jkrs\cdot} - \bar{y}^*_{....})^2$$

REFERENCES

Wang, T. (2014), A revised fisher model on analysis of quantitative trait loci with multiple alleles., *Front Genet*, 5, 328, doi:10.3389/fgene.2014.00328
